# Supplementary material for: Transposable elements drive intron gain in diverse eukaryotes
Source: Proc Natl Acad Sci U S A. 2022 Nov 23;119(48):e2209766119. doi: 10.1073/pnas.2209766119 (PMC9860276; doi:10.1073/pnas.2209766119)
Supplement: Supplementary file 1 — Appendix 01 (PDF) [file pnas.2209766119.sapp.pdf]

Supplementary Figures

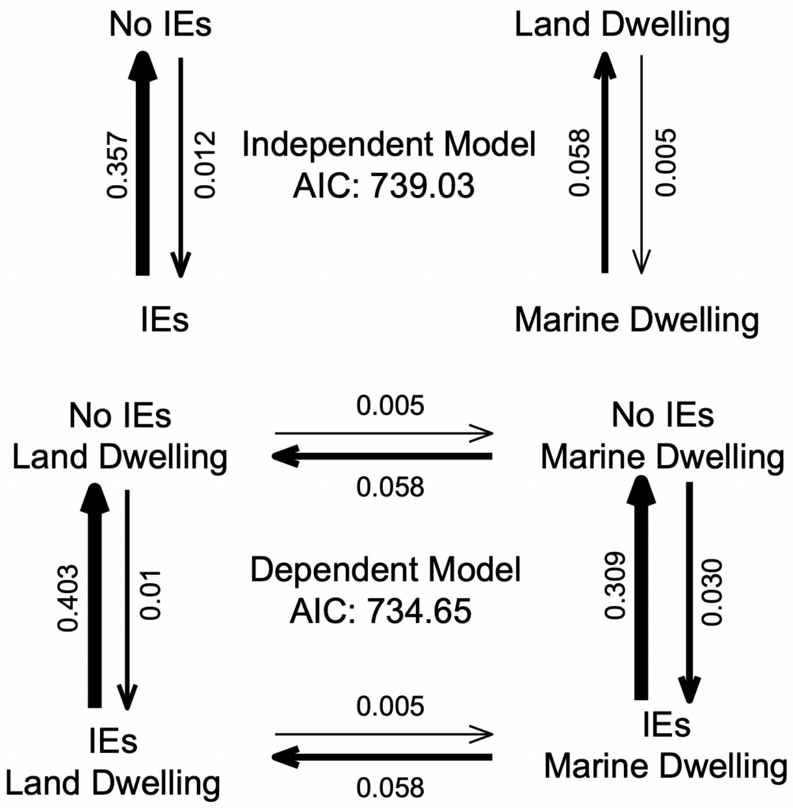

Supplementary Figure 1: The model in which Introner evolution is dependent on whether or not a species is marine dwelling is a better fit to our data than a model in which the two traits evolve independently. We used Pagel's test to calculate the transition rates for four models (independent, Introner presence dependent on germline accessibility, germline accessibility dependent on Introner presence, and co-dependent) with the former two shown here. Based on the AIC, the dependent model better fits our data ($P < 4.1\times10^{-4}$).

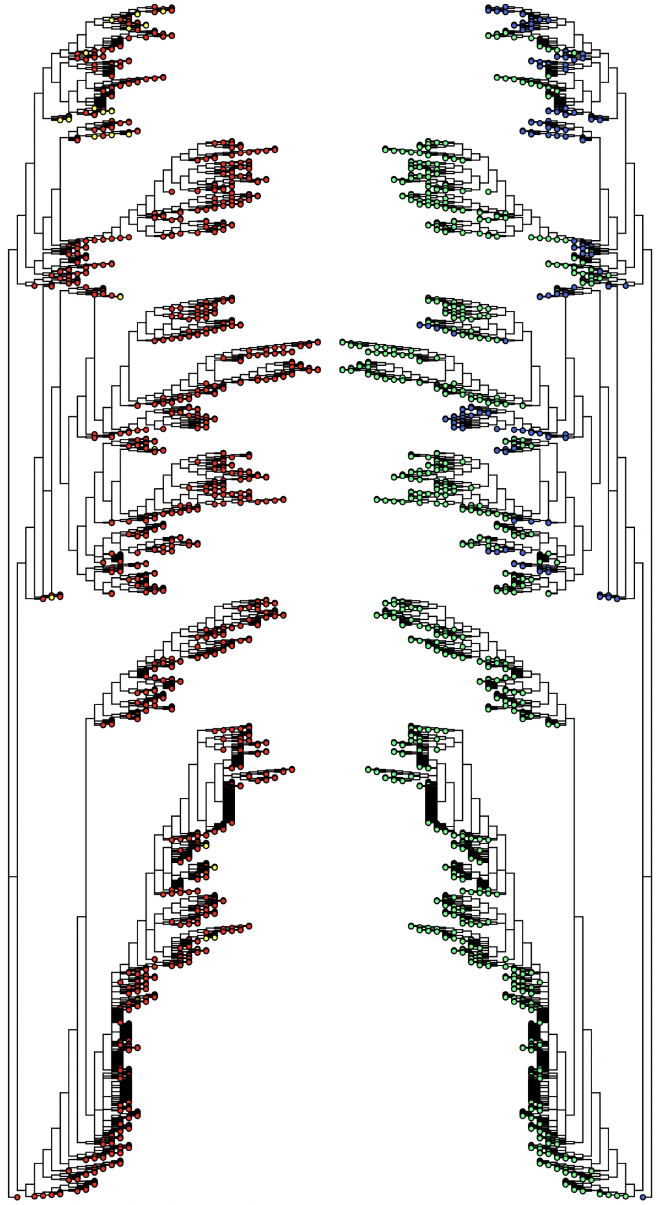

Supplementary Figure 2: Tree for association with aquatic species. The phylogeny used to test for association between the presence of Introners and aquatic organisms is shown with marine dwelling species in blue and land dwelling species in green (right), and with Introner-containing species in yellow and those without Introners in red (left). The phylogeny is mirrored for comparisons between the two sets of traits.

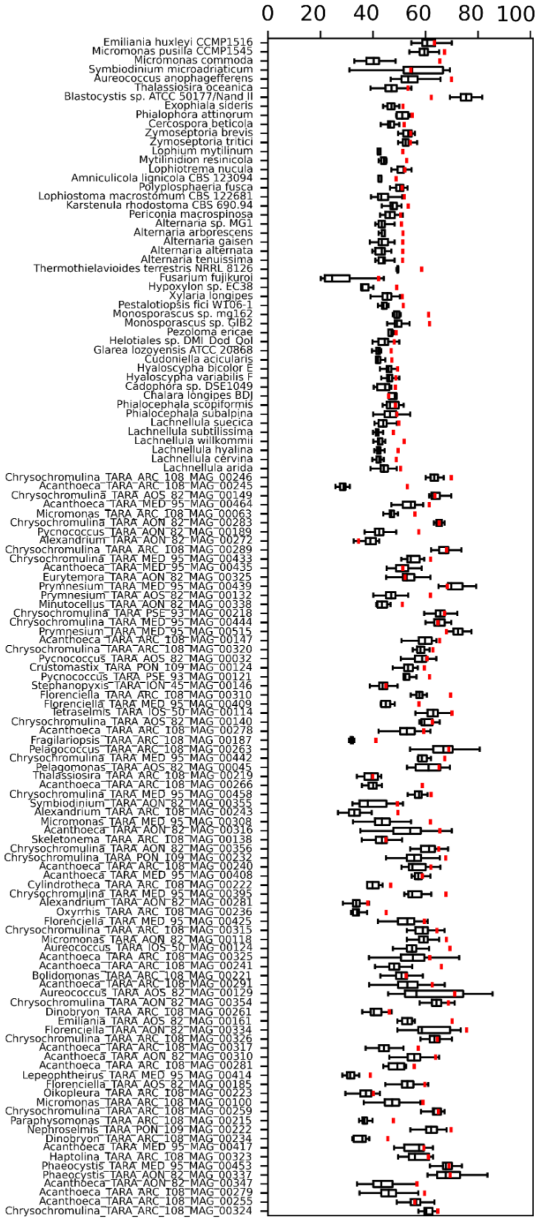

Supplementary Figure 3: Insertion of Introners into GC rich regions. Box plots represent 1000 permutations for the GC content of possible insertion sites within Introner containing genes as determined by randomization. Red bars denote GC content of the 10bp surrounding observed insertion sites.

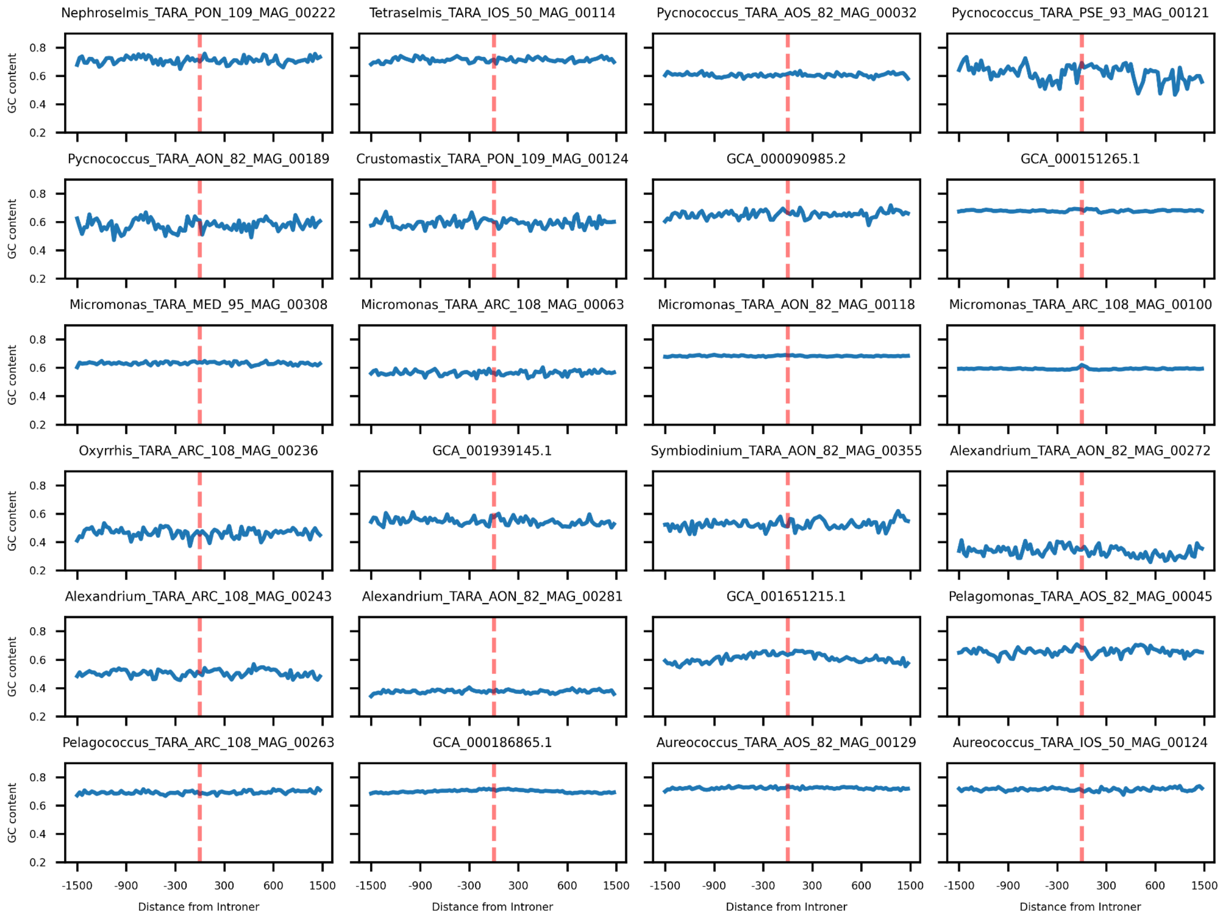
Nephroselmis_TARA_PON_109_MAG_00222
Tetraselmis_TARA_IOS_50_MAG_00114
Pycnococcus_TARA_AOS_82_MAG_00032
Pycnococcus_TARA_PSE_93_MAG_00121
Pycnococcus_TARA_AON_82_MAG_00189
Crustomastix_TARA_PON_109_MAG_00124
GCA_000090985.2
GCA_000151265.1
Micromonas_TARA_MED_95_MAG_00308
Micromonas_TARA_ARC_108_MAG_00063
Micromonas_TARA_AON_82_MAG_00118
Micromonas_TARA_ARC_108_MAG_00100
Oxyrrhis_TARA_ARC_108_MAG_00236
GCA_001939145.1
Symbiodinium_TARA_AON_82_MAG_00355
Alexandrium_TARA_AON_82_MAG_00272
Alexandrium_TARA_ARC_108_MAG_00243
Alexandrium_TARA_AON_82_MAG_00281
GCA_001651215.1
Pelagomonas_TARA_AOS_82_MAG_00045
Pelagococcus_TARA_ARC_108_MAG_00263
GCA_000186865.1
Aureococcus_TARA_AOS_82_MAG_00129
Aureococcus_TARA_IOS_50_MAG_00124
GC content
0.2
0.4
0.6
0.8
-1500
-900
-300
300
600
1500
Distance from Introner

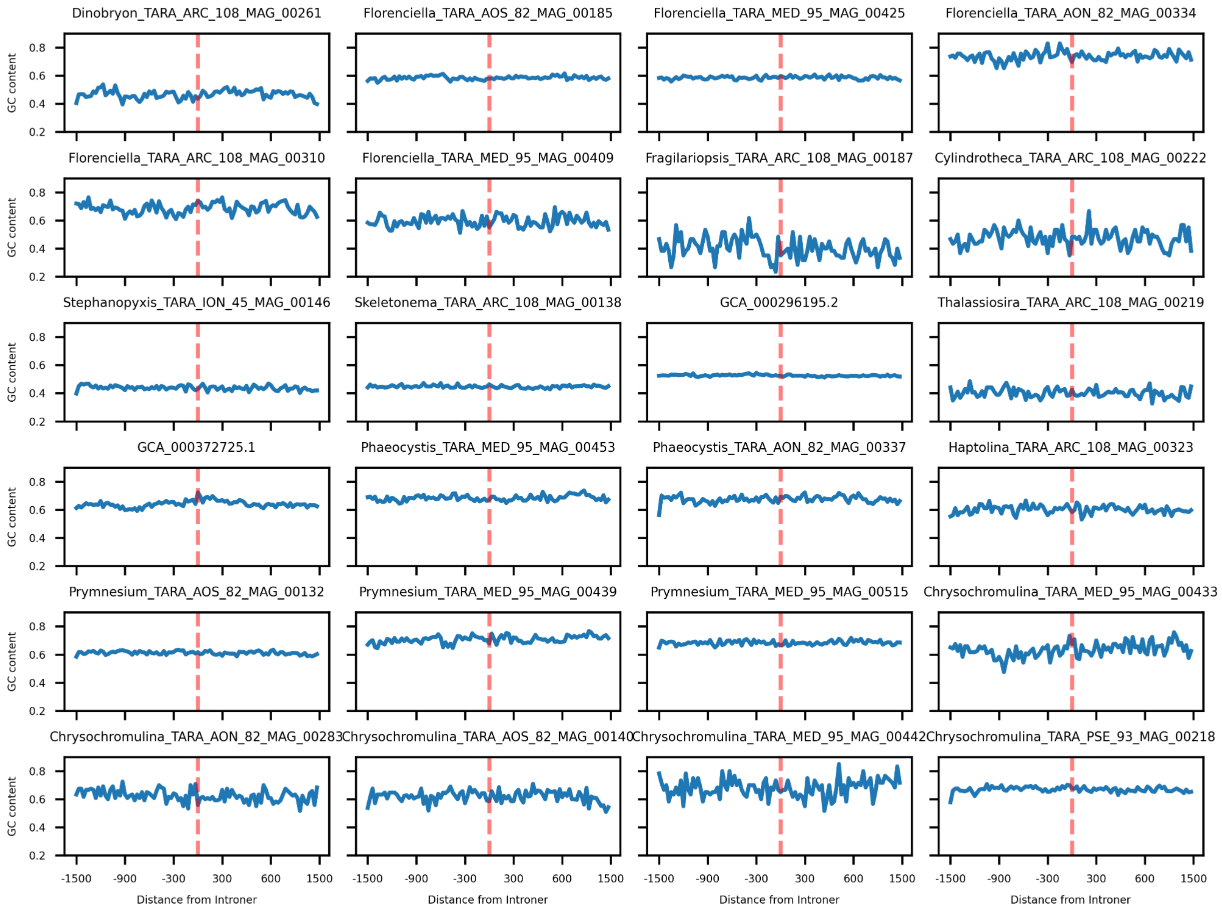
Dinobryon_TARA_ARC_108_MAG_00261
Florenciella_TARA_AOS_82_MAG_00185
Florenciella_TARA_MED_95_MAG_00425
Florenciella_TARA_AON_82_MAG_00334
Florenciella_TARA_ARC_108_MAG_00310
Florenciella_TARA_MED_95_MAG_00409
Fragilariopsis_TARA_ARC_108_MAG_00187
Cylindrotheca_TARA_ARC_108_MAG_00222
Stephanopyxis_TARA_ION_45_MAG_00146
Skeletonema_TARA_ARC_108_MAG_00138
GCA_000296195.2
Thalassiosira_TARA_ARC_108_MAG_00219
GCA_000372725.1
Phaeocystis_TARA_MED_95_MAG_00453
Phaeocystis_TARA_AON_82_MAG_00337
Haptolina_TARA_ARC_108_MAG_00323
Prymnesium_TARA_AOS_82_MAG_00132
Prymnesium_TARA_MED_95_MAG_00439
Prymnesium_TARA_MED_95_MAG_00515
Chrysochromulina_TARA_MED_95_MAG_00433
Chrysochromulina_TARA_AON_82_MAG_00283
Chrysochromulina_TARA_AOS_82_MAG_00140
Chrysochromulina_TARA_MED_95_MAG_00442
Chrysochromulina_TARA_PSE_93_MAG_00218
GC content
0.8
0.6
0.4
0.2
-1500
-900
-300
300
600
1500
Distance from Introner

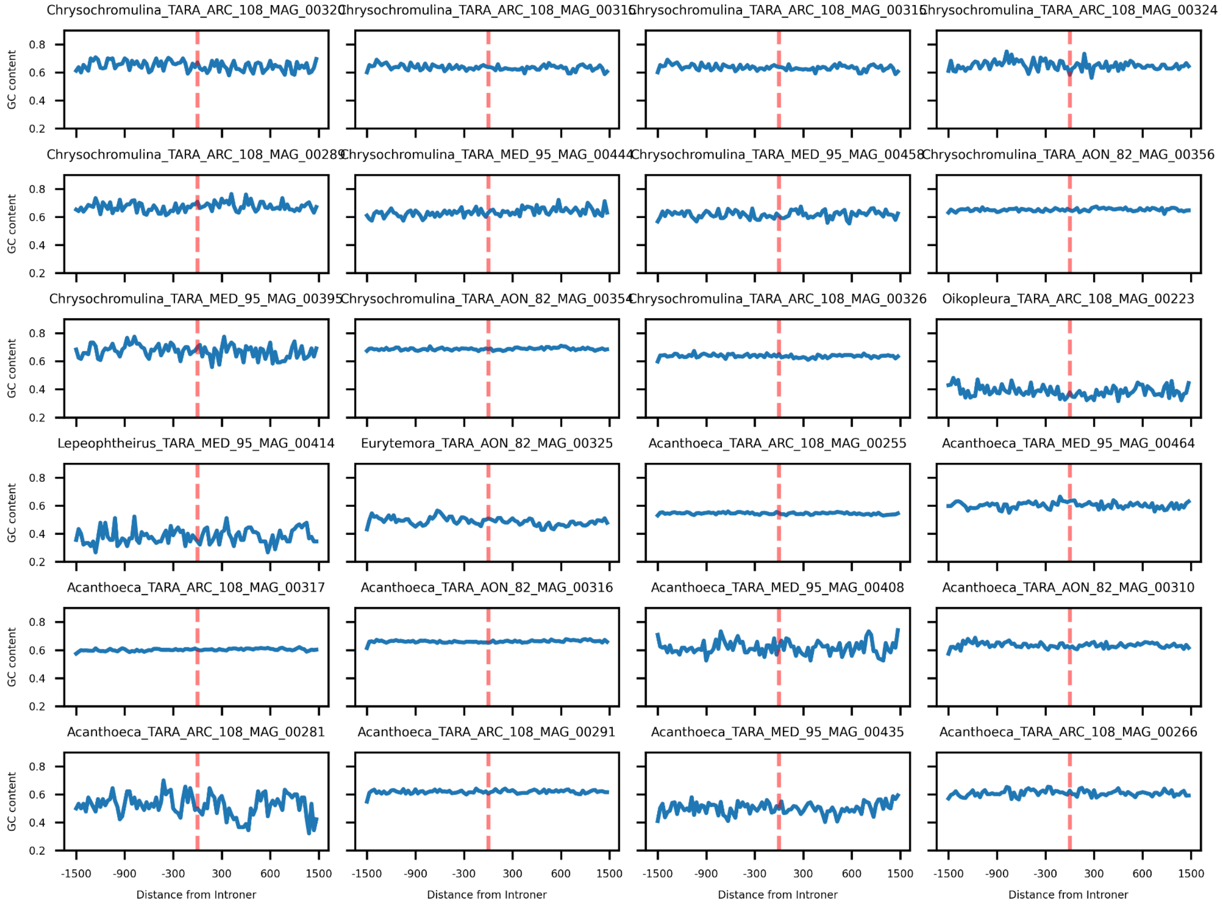

Chrysochromulina_TARA_ARC_108_MAG_00320
Chrysochromulina_TARA_ARC_108_MAG_00316
Chrysochromulina_TARA_ARC_108_MAG_00315
Chrysochromulina_TARA_ARC_108_MAG_00324
Chrysochromulina_TARA_ARC_108_MAG_00289
Chrysochromulina_TARA_MED_95_MAG_00444
Chrysochromulina_TARA_MED_95_MAG_00458
Chrysochromulina_TARA_AON_82_MAG_00356
Chrysochromulina_TARA_MED_95_MAG_00395
Chrysochromulina_TARA_AON_82_MAG_00354
Chrysochromulina_TARA_ARC_108_MAG_00326
Oikopleura_TARA_ARC_108_MAG_00223
Lepeophtheirus_TARA_MED_95_MAG_00414
Eurytemora_TARA_AON_82_MAG_00325
Acanthoeca_TARA_ARC_108_MAG_00255
Acanthoeca_TARA_MED_95_MAG_00464
Acanthoeca_TARA_ARC_108_MAG_00317
Acanthoeca_TARA_AON_82_MAG_00316
Acanthoeca_TARA_MED_95_MAG_00408
Acanthoeca_TARA_AON_82_MAG_00310
Acanthoeca_TARA_ARC_108_MAG_00281
Acanthoeca_TARA_ARC_108_MAG_00291
Acanthoeca_TARA_MED_95_MAG_00435
Acanthoeca_TARA_ARC_108_MAG_00266
GC content
0.2
0.4
0.6
0.8
-1500
-900
-300
300
600
1500
Distance from Introner

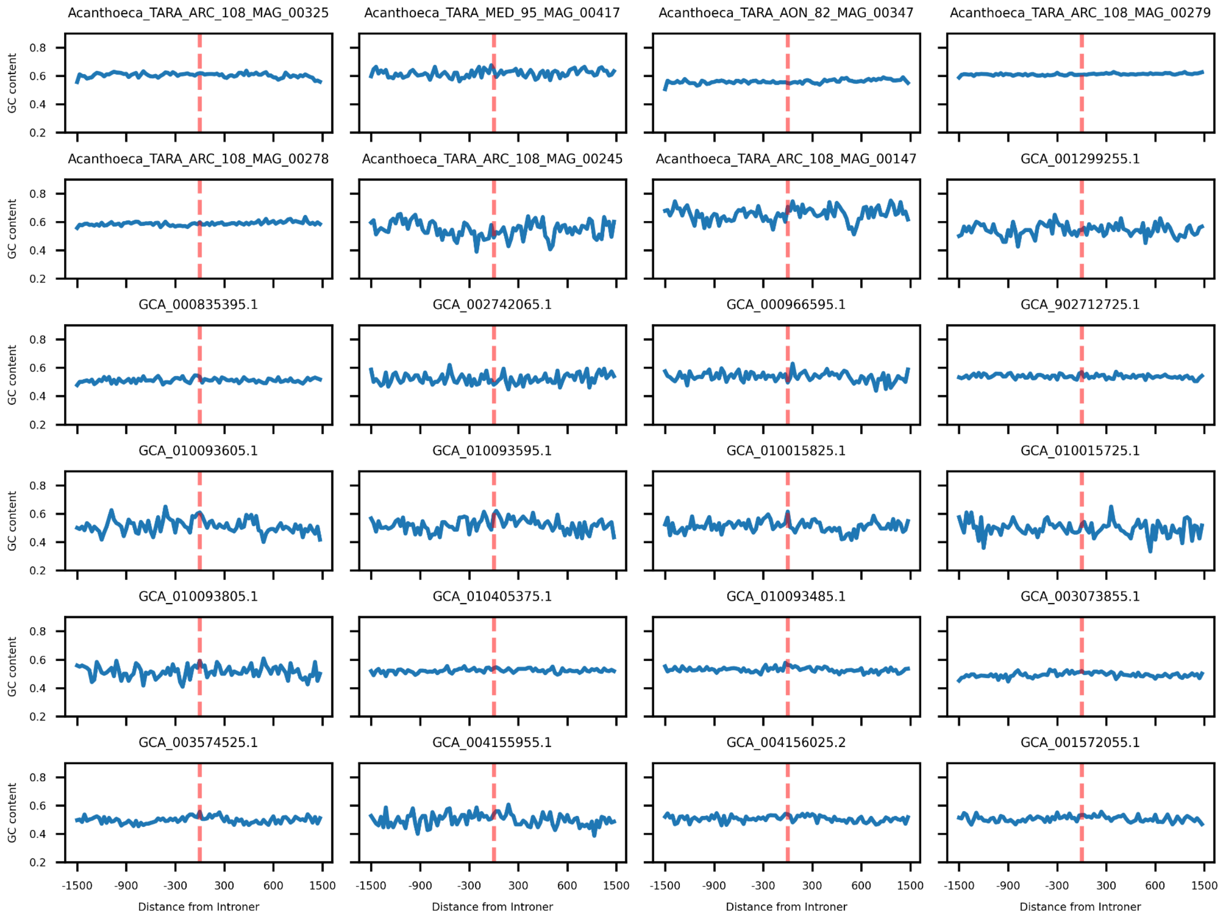
Acanthoeca_TARA_ARC_108_MAG_00325
Acanthoeca_TARA_MED_95_MAG_00417
Acanthoeca_TARA_AON_82_MAG_00347
Acanthoeca_TARA_ARC_108_MAG_00279
Acanthoeca_TARA_ARC_108_MAG_00278
Acanthoeca_TARA_ARC_108_MAG_00245
Acanthoeca_TARA_ARC_108_MAG_00147
GCA_001299255.1
GCA_000835395.1
GCA_002742065.1
GCA_000966595.1
GCA_902712725.1
GCA_010093605.1
GCA_010093595.1
GCA_010015825.1
GCA_010015725.1
GCA_010093805.1
GCA_010405375.1
GCA_010093485.1
GCA_003073855.1
GCA_003574525.1
GCA_004155955.1
GCA_004156025.2
GCA_001572055.1
GC content
0.2
0.4
0.6
0.8
-1500
-900
-300
300
600
1500
Distance from Introner

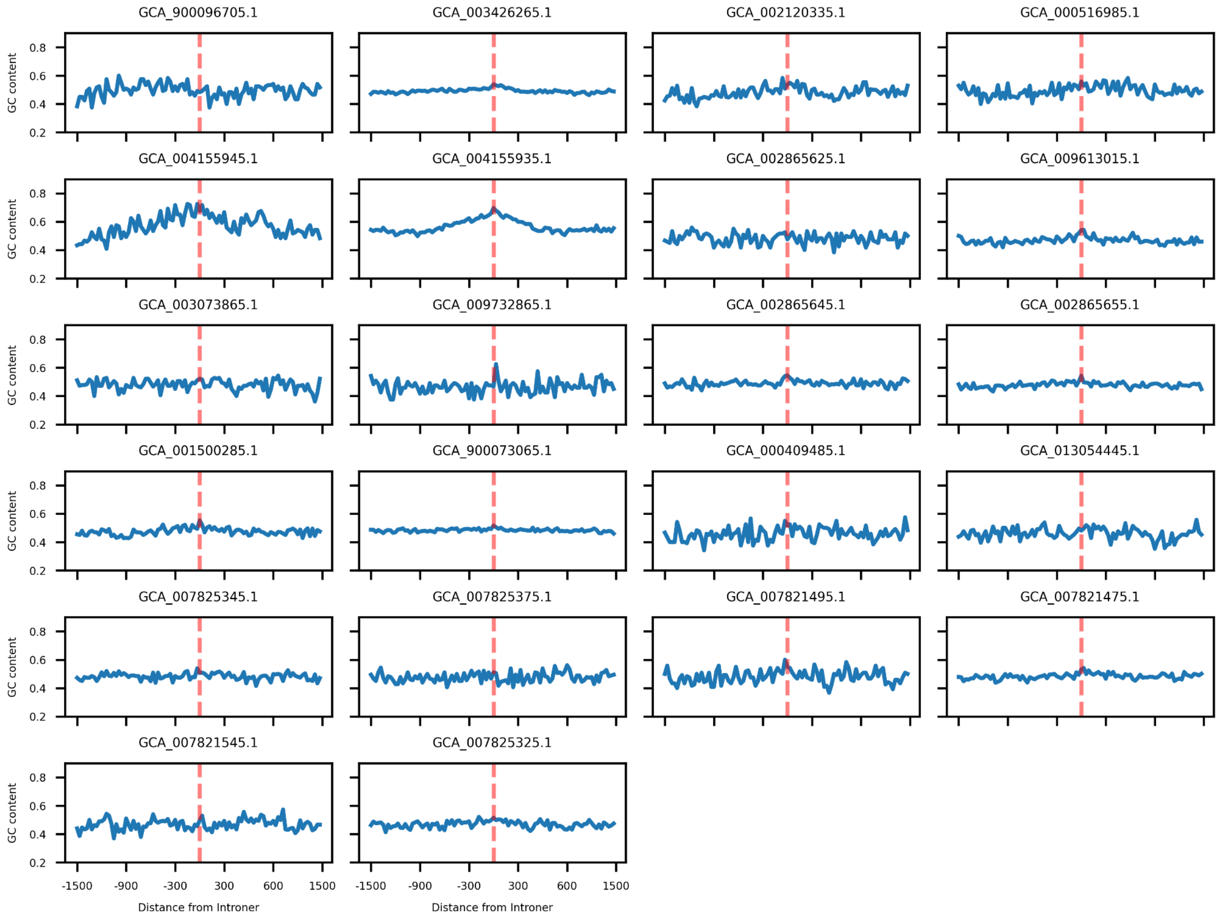

Supplementary Figure 4: GC content around Introner insertion sites. Plots show the average GC content calculated for 30bp windows around Introner insertion sites (with Introners removed), with the X axis showing distance from Introners in bp. Introner insertion sites are represented by the red dotted line. Negative values on the X axis represent distance upstream with respect to the position of Introners. Plots were generated for genomes in which we were able to extract the 3000bp surrounding Introner insertion sites for at least 2 Introners.

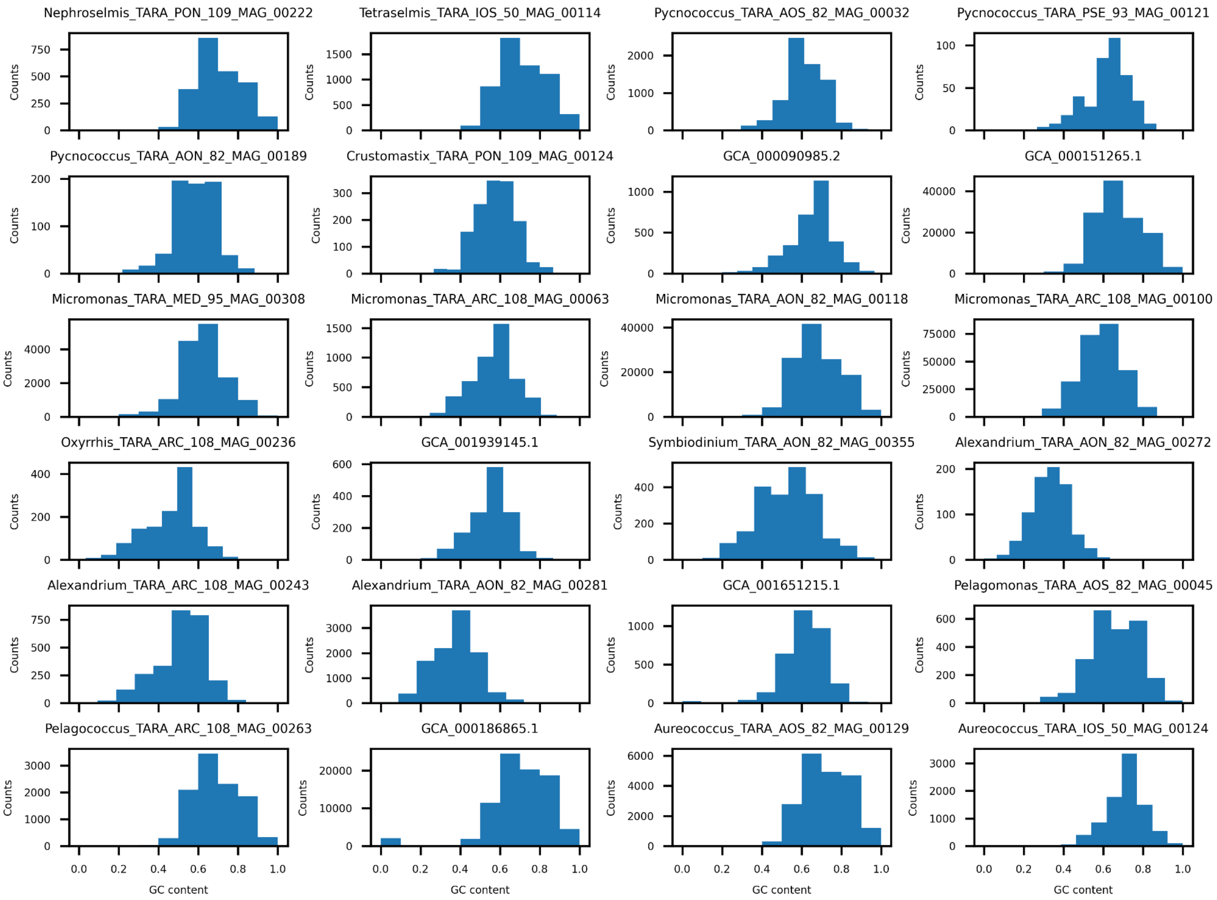

Nephroselmis_TARA_PON_109_MAG_00222
Tetraselmis_TARA_IOS_50_MAG_00114
Pycnococcus_TARA_AOS_82_MAG_00032
Pycnococcus_TARA_PSE_93_MAG_00121
Pycnococcus_TARA_AON_82_MAG_00189
Crustomastix_TARA_PON_109_MAG_00124
GCA_000090985.2
GCA_000151265.1
Micromonas_TARA_MED_95_MAG_00308
Micromonas_TARA_ARC_108_MAG_00063
Micromonas_TARA_AON_82_MAG_00118
Micromonas_TARA_ARC_108_MAG_00100
Oxyrrhis_TARA_ARC_108_MAG_00236
GCA_001939145.1
Symbiodinium_TARA_AON_82_MAG_00355
Alexandrium_TARA_AON_82_MAG_00272
Alexandrium_TARA_ARC_108_MAG_00243
Alexandrium_TARA_AON_82_MAG_00281
GCA_001651215.1
Pelagomonas_TARA_AOS_82_MAG_00045
Pelagococcus_TARA_ARC_108_MAG_00263
GCA_000186865.1
Aureococcus_TARA_AOS_82_MAG_00129
Aureococcus_TARA_IOS_50_MAG_00124
Counts
GC content
0.0
0.2
0.4
0.6
0.8
1.0

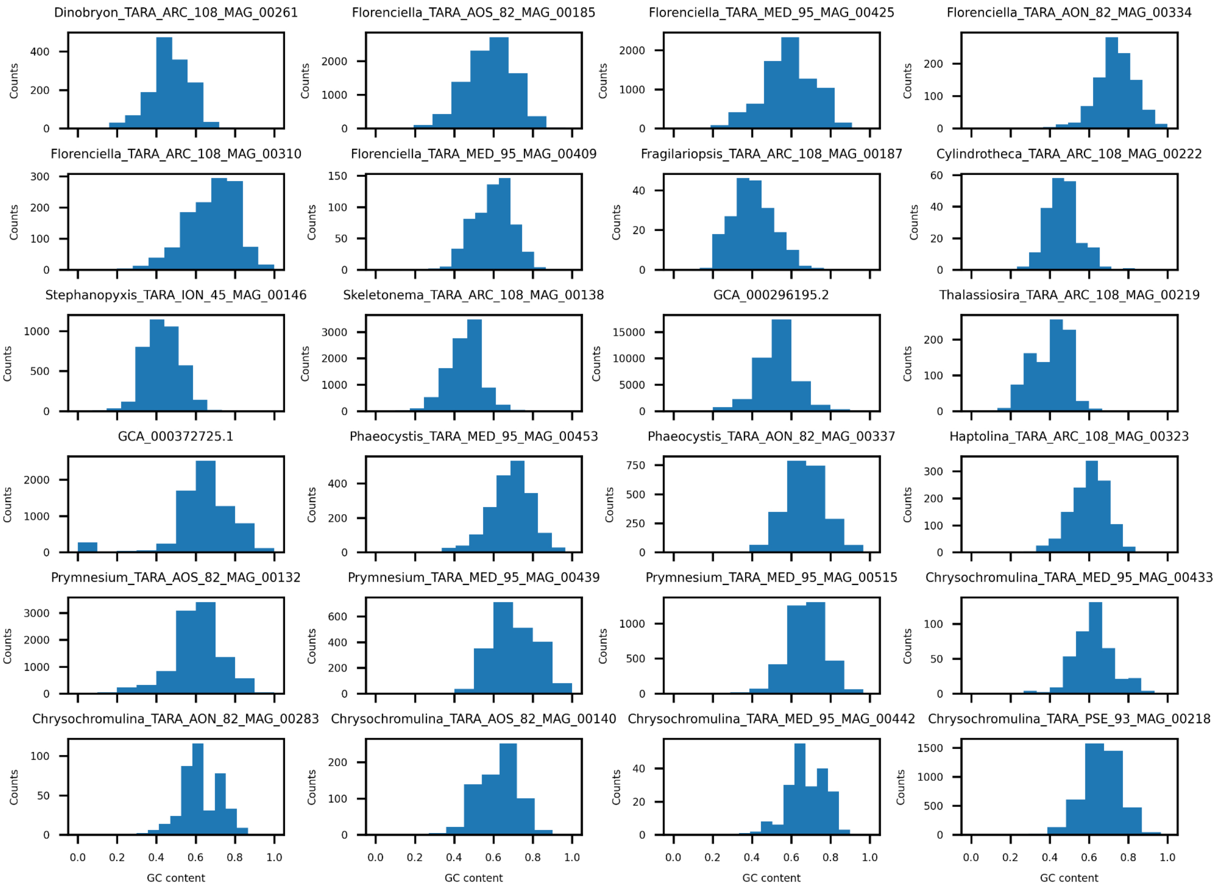
Dinobryon_TARA_ARC_108_MAG_00261
Florenciella_TARA_AOS_82_MAG_00185
Florenciella_TARA_MED_95_MAG_00425
Florenciella_TARA_AON_82_MAG_00334
Florenciella_TARA_ARC_108_MAG_00310
Florenciella_TARA_MED_95_MAG_00409
Fragilariopsis_TARA_ARC_108_MAG_00187
Cylindrotheca_TARA_ARC_108_MAG_00222
Stephanopyxis_TARA_ION_45_MAG_00146
Skeletonema_TARA_ARC_108_MAG_00138
GCA_000296195.2
Thalassiosira_TARA_ARC_108_MAG_00219
GCA_000372725.1
Phaeocystis_TARA_MED_95_MAG_00453
Phaeocystis_TARA_AON_82_MAG_00337
Haptolina_TARA_ARC_108_MAG_00323
Prymnesium_TARA_AOS_82_MAG_00132
Prymnesium_TARA_MED_95_MAG_00439
Prymnesium_TARA_MED_95_MAG_00515
Chrysochromulina_TARA_MED_95_MAG_00433
Chrysochromulina_TARA_AON_82_MAG_00283
Chrysochromulina_TARA_AOS_82_MAG_00140
Chrysochromulina_TARA_MED_95_MAG_00442
Chrysochromulina_TARA_PSE_93_MAG_00218
Counts
GC content
0.0
0.2
0.4
0.6
0.8
1.0

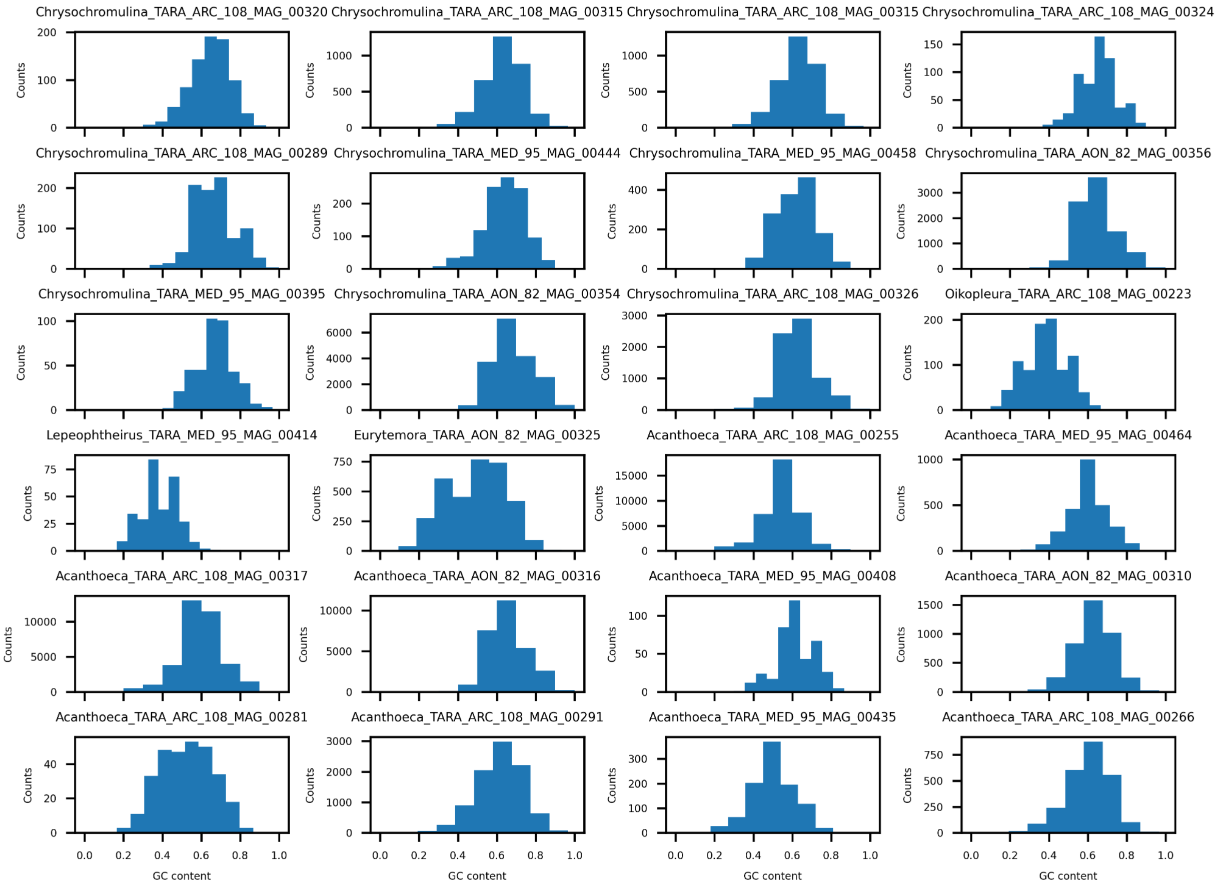
Chrysochromulina_TARA_ARC_108_MAG_00320
Chrysochromulina_TARA_ARC_108_MAG_00315
Chrysochromulina_TARA_ARC_108_MAG_00315
Chrysochromulina_TARA_ARC_108_MAG_00324
Chrysochromulina_TARA_ARC_108_MAG_00289
Chrysochromulina_TARA_MED_95_MAG_00444
Chrysochromulina_TARA_MED_95_MAG_00458
Chrysochromulina_TARA_AON_82_MAG_00356
Chrysochromulina_TARA_MED_95_MAG_00395
Chrysochromulina_TARA_AON_82_MAG_00354
Chrysochromulina_TARA_ARC_108_MAG_00326
Oikopleura_TARA_ARC_108_MAG_00223
Lepeophtheirus_TARA_MED_95_MAG_00414
Eurytemora_TARA_AON_82_MAG_00325
Acanthoeca_TARA_ARC_108_MAG_00255
Acanthoeca_TARA_MED_95_MAG_00464
Acanthoeca_TARA_ARC_108_MAG_00317
Acanthoeca_TARA_AON_82_MAG_00316
Acanthoeca_TARA_MED_95_MAG_00408
Acanthoeca_TARA_AON_82_MAG_00310
Acanthoeca_TARA_ARC_108_MAG_00281
Acanthoeca_TARA_ARC_108_MAG_00291
Acanthoeca_TARA_MED_95_MAG_00435
Acanthoeca_TARA_ARC_108_MAG_00266
Counts
0.0 0.2 0.4 0.6 0.8 1.0
GC content

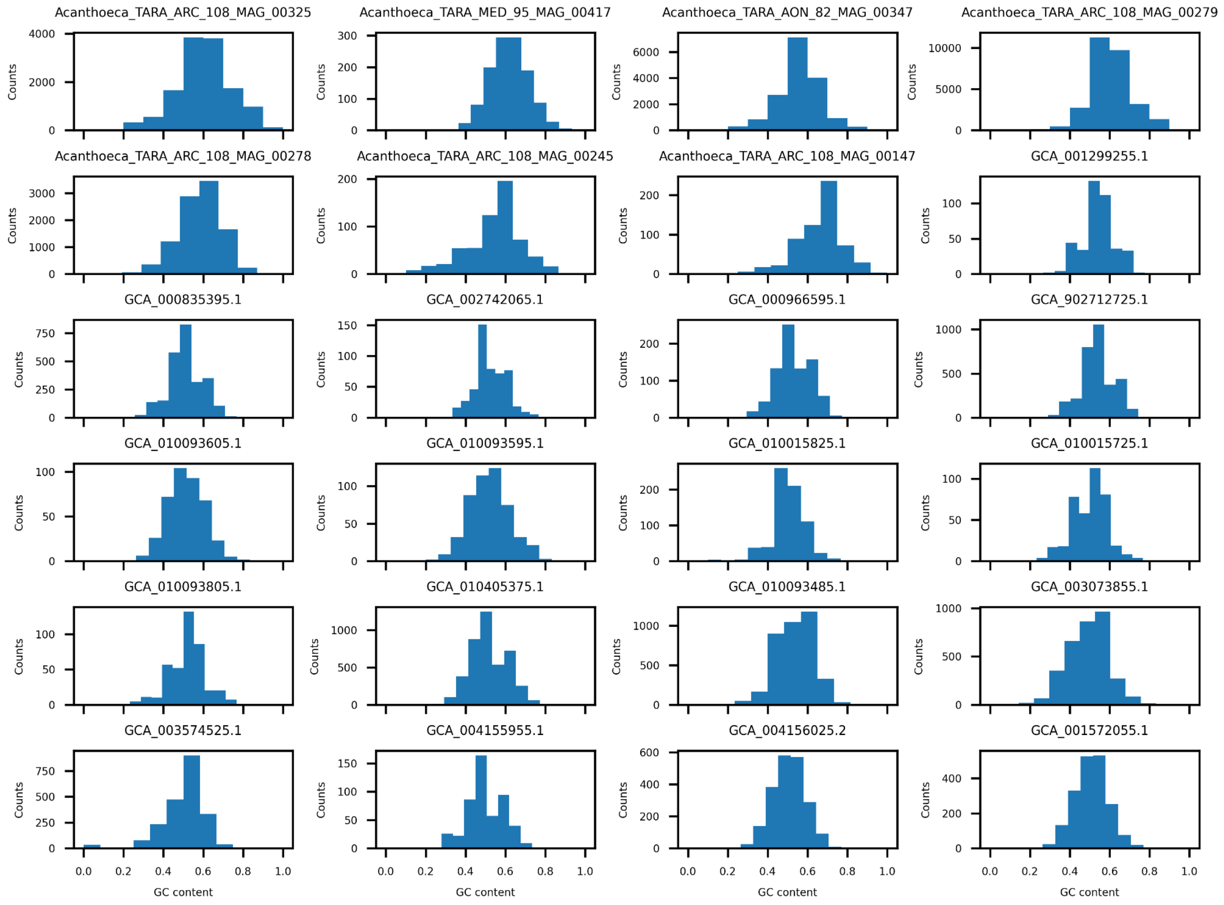
Acanthoeca_TARA_ARC_108_MAG_00325
Acanthoeca_TARA_MED_95_MAG_00417
Acanthoeca_TARA_AON_82_MAG_00347
Acanthoeca_TARA_ARC_108_MAG_00279
Acanthoeca_TARA_ARC_108_MAG_00278
Acanthoeca_TARA_ARC_108_MAG_00245
Acanthoeca_TARA_ARC_108_MAG_00147
GCA_001299255.1
GCA_000835395.1
GCA_002742065.1
GCA_000966595.1
GCA_902712725.1
GCA_010093605.1
GCA_010093595.1
GCA_010015825.1
GCA_010015725.1
GCA_010093805.1
GCA_010405375.1
GCA_010093485.1
GCA_003073855.1
GCA_003574525.1
GCA_004155955.1
GCA_004156025.2
GCA_001572055.1
Counts
0.0 0.2 0.4 0.6 0.8 1.0
GC content

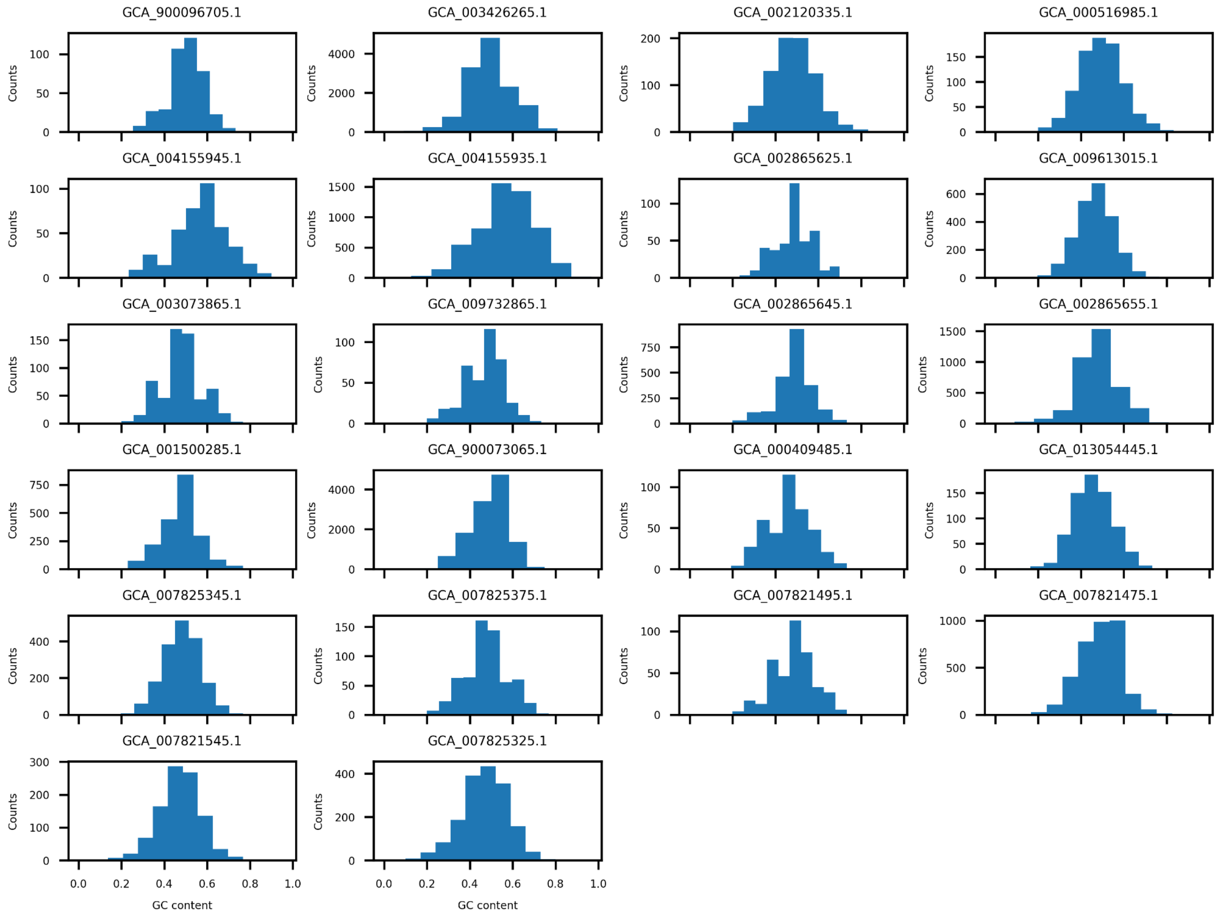

Supplementary Figure 5: Distribution of GC content surrounding Introner insertions. Histograms display the aggregated distribution of GC content calculated across 30 bp windows for the 3000 bp surrounding Introner insertions for each species (1500 bp upstream and 1500 bp downstream).

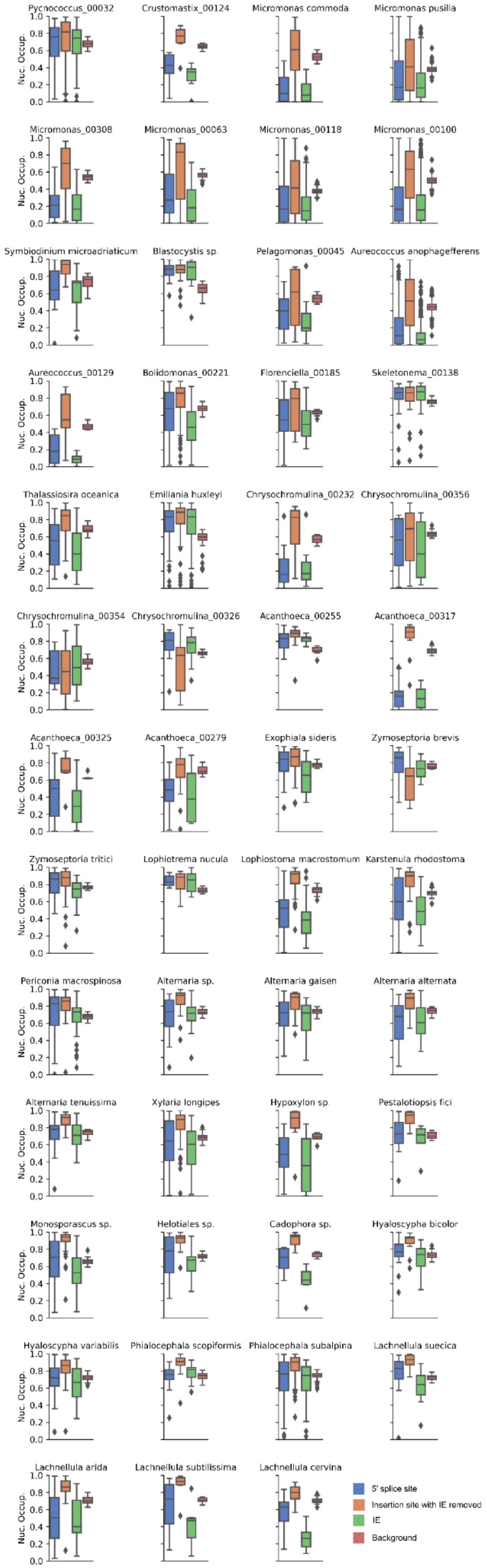
Pycnococcus_00032
Crustomastix_00124
Micromonas commoda
Micromonas pusilla
Micromonas_00308
Micromonas_00063
Micromonas_00118
Micromonas_00100
Symbiodinium microadriaticum
Blastocystis sp.
Pelagomonas_00045
Aureococcus anophagefferens
Aureococcus_00129
Bolidomonas_00221
Florenciella_00185
Skeletonema_00138
Thalassiosira oceanica
Emiliania huxleyi
Chrysochromulina_00232
Chrysochromulina_00356
Chrysochromulina_00354
Chrysochromulina_00326
Acanthoeca_00255
Acanthoeca_00317
Acanthoeca_00325
Acanthoeca_00279
Exophiala sideris
Zymoseptoria brevis
Zymoseptoria tritici
Lophiotrema nucula
Lophiostoma macrostomum
Karstenula rhodostoma
Periconia macrospinosa
Alternaria sp.
Alternaria gaisen
Alternaria alternata
Alternaria tenuissima
Xylaria longipes
Hypoxylon sp.
Pestalotiopsis fici
Monosporascus sp.
Helotiales sp.
Cadophora sp.
Hyaloscypha bicolor
Hyaloscypha variabilis
Phialocephala scopiformis
Phialocephala subalpina
Lachnellula suecica
Lachnellula arida
Lachnellula subtilissima
Lachnellula cervina
Nuc. Occup.
5' splice site
Insertion site with IE removed
IE
Background

Supplementary Figure 6: Nucleosome occupancy with Introners present and Introners removed. Box plots represent predicted nucleosome occupancy distributions for different regions related to Introners for all Introners in each species. Colors denote the region, with blue showing the nucleosome occupancy for the 100bp surrounding the 5' splice site, orange showing nucleosome occupancy for the 100bp surrounding the Introner with the Introner removed, green showing the nucleosome occupancy across Introner sequences, and red showing the background nucleosome occupancy. In most species, we find that the 100bp surrounding the 5' splice site of Introners exhibits lower nucleosome occupancy relative to background regions (p = 8.26e-07; binomial). We observe the same pattern for Introner sequences (p = 4.00e-10; binomial). When we remove Introners, we instead observe the opposite pattern, in which nucleosome occupancy is high relative to the background, suggesting that Introners nucleosomes are often bordered by nucleosomes (p = 9.13e-12; binomial).

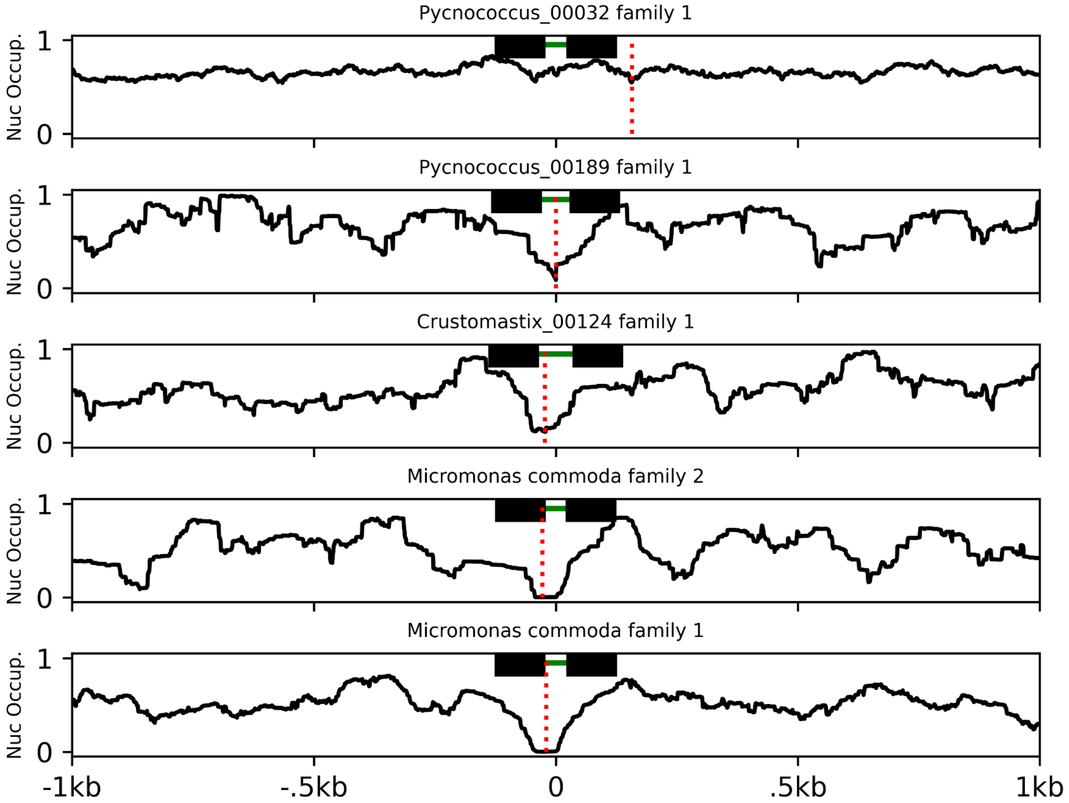

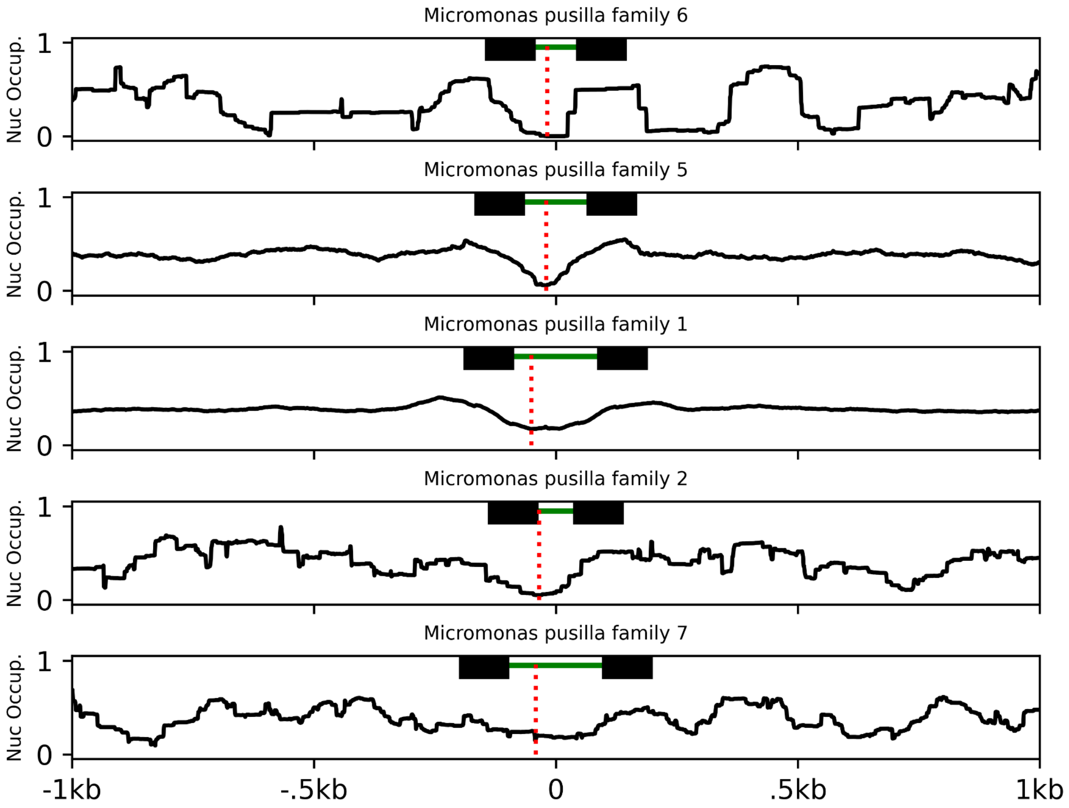
Micromonas pusilla family 6
Micromonas pusilla family 5
Micromonas pusilla family 1
Micromonas pusilla family 2
Micromonas pusilla family 7
Nuc Occup.
1
0
-1kb
-.5kb
0
.5kb
1kb

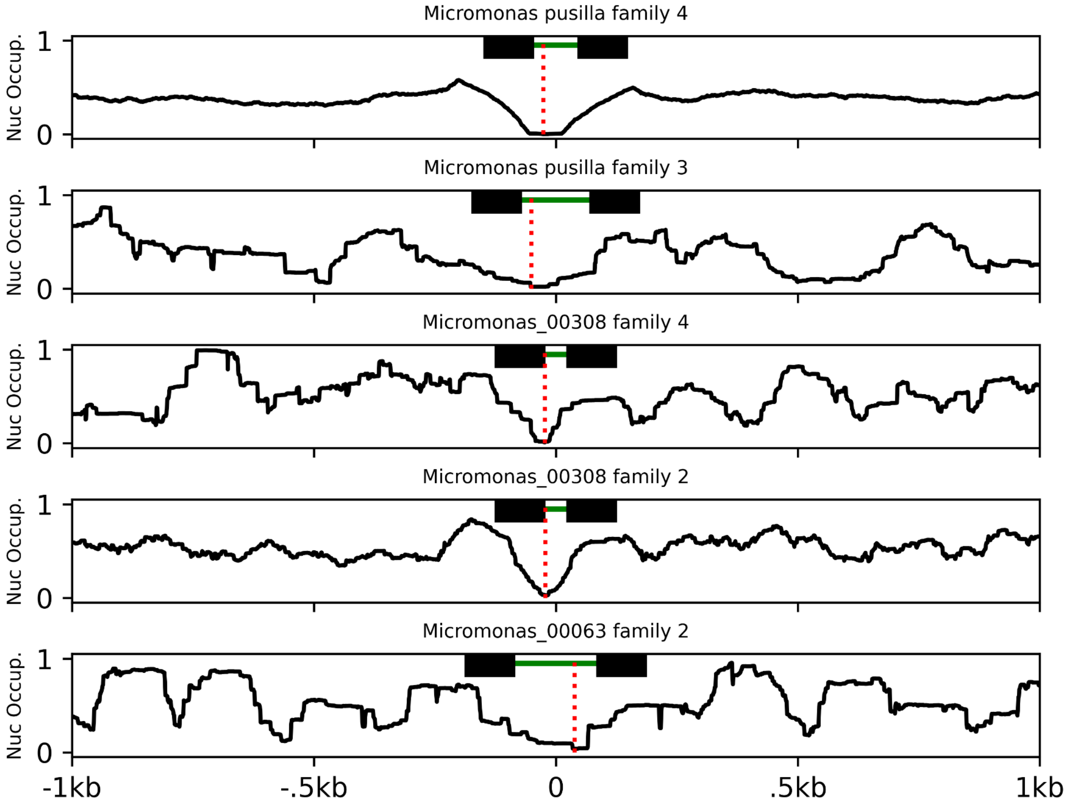

Micromonas pusilla family 4
Micromonas pusilla family 3
Micromonas_00308 family 4
Micromonas_00308 family 2
Micromonas_00063 family 2
Nuc Occup.
1
0
-1kb
-.5kb
0
.5kb
1kb

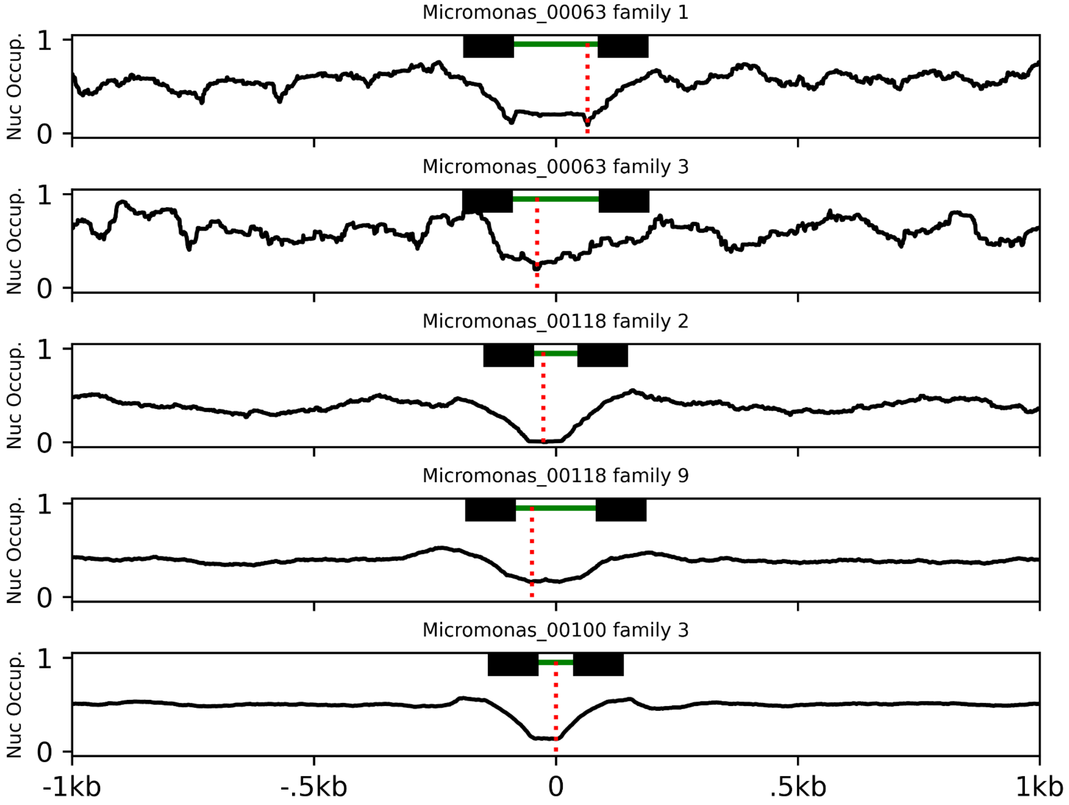

Micromonas_00063 family 1
Micromonas_00063 family 3
Micromonas_00118 family 2
Micromonas_00118 family 9
Micromonas_00100 family 3
Nuc Occup.
1
0
-1kb
-.5kb
0
.5kb
1kb

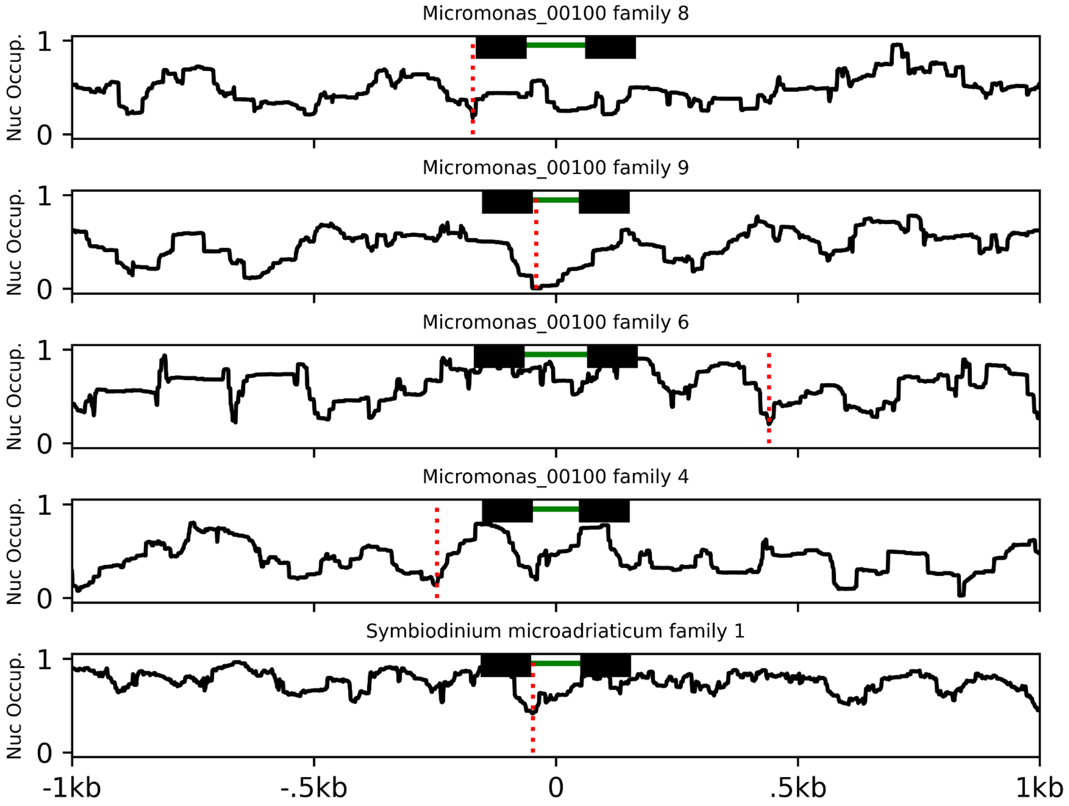

Micromonas_00100 family 8
Nuc Occup.
1
0
Micromonas_00100 family 9
Nuc Occup.
1
0
Micromonas_00100 family 6
Nuc Occup.
1
0
Micromonas_00100 family 4
Nuc Occup.
1
0
Symbiodinium microadriaticum family 1
Nuc Occup.
1
0
-1kb
-.5kb
0
.5kb
1kb

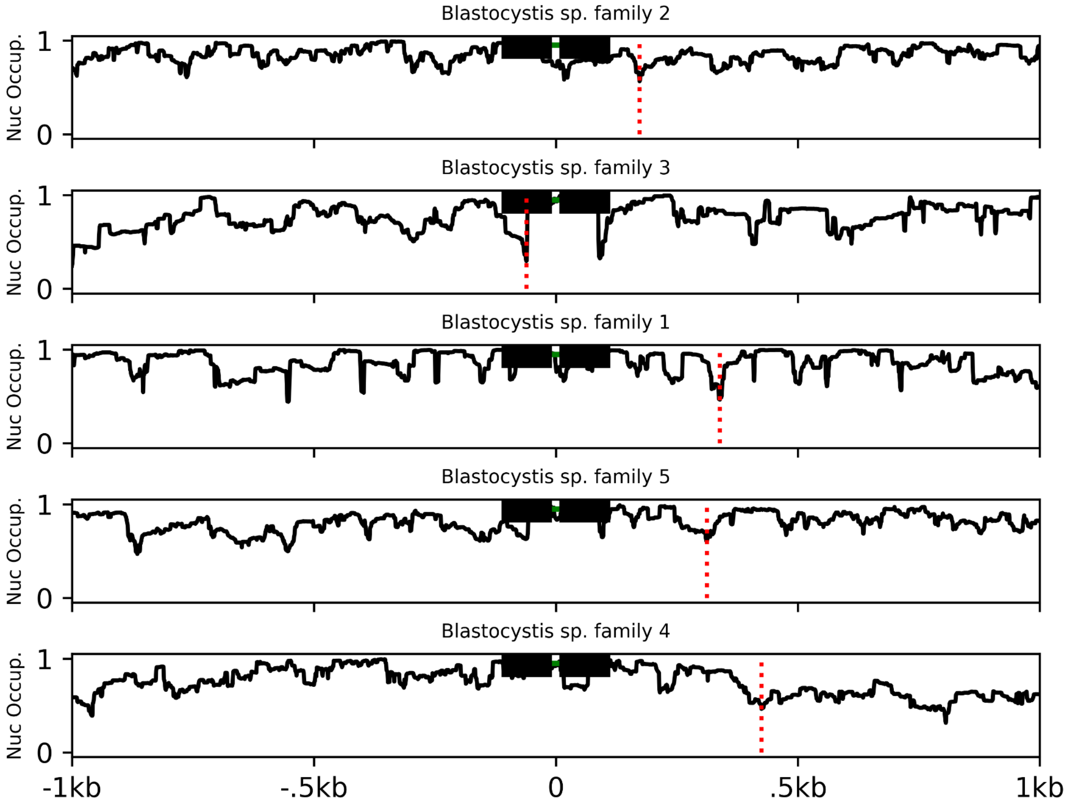

Blastocystis sp. family 2
Blastocystis sp. family 3
Blastocystis sp. family 1
Blastocystis sp. family 5
Blastocystis sp. family 4
Nuc Occup.
1
0
-1kb
-.5kb
0
.5kb
1kb

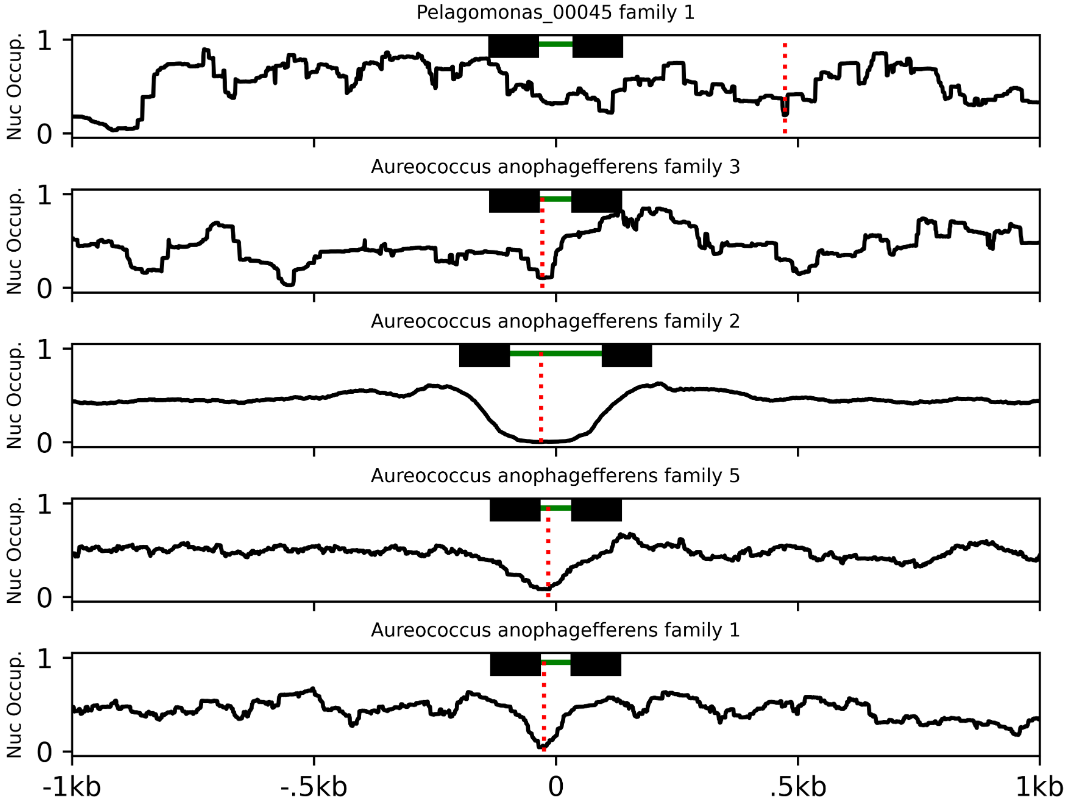
Pelagomonas_00045 family 1
Aureococcus anophagefferens family 3
Aureococcus anophagefferens family 2
Aureococcus anophagefferens family 5
Aureococcus anophagefferens family 1
Nuc Occup.
1
0
-1kb
-.5kb
0
.5kb
1kb

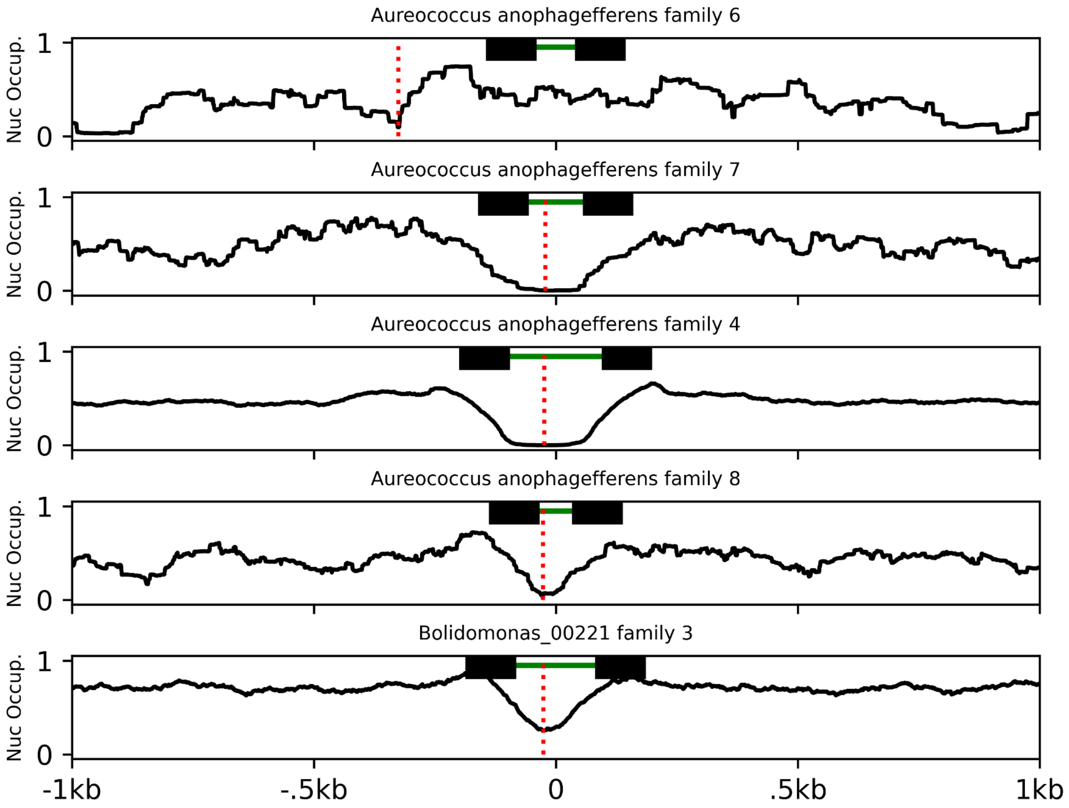
Aureococcus anophagefferens family 6
Aureococcus anophagefferens family 7
Aureococcus anophagefferens family 4
Aureococcus anophagefferens family 8
Bolidomonas_00221 family 3
Nuc Occup.
1
0
-1kb
-.5kb
0
.5kb
1kb

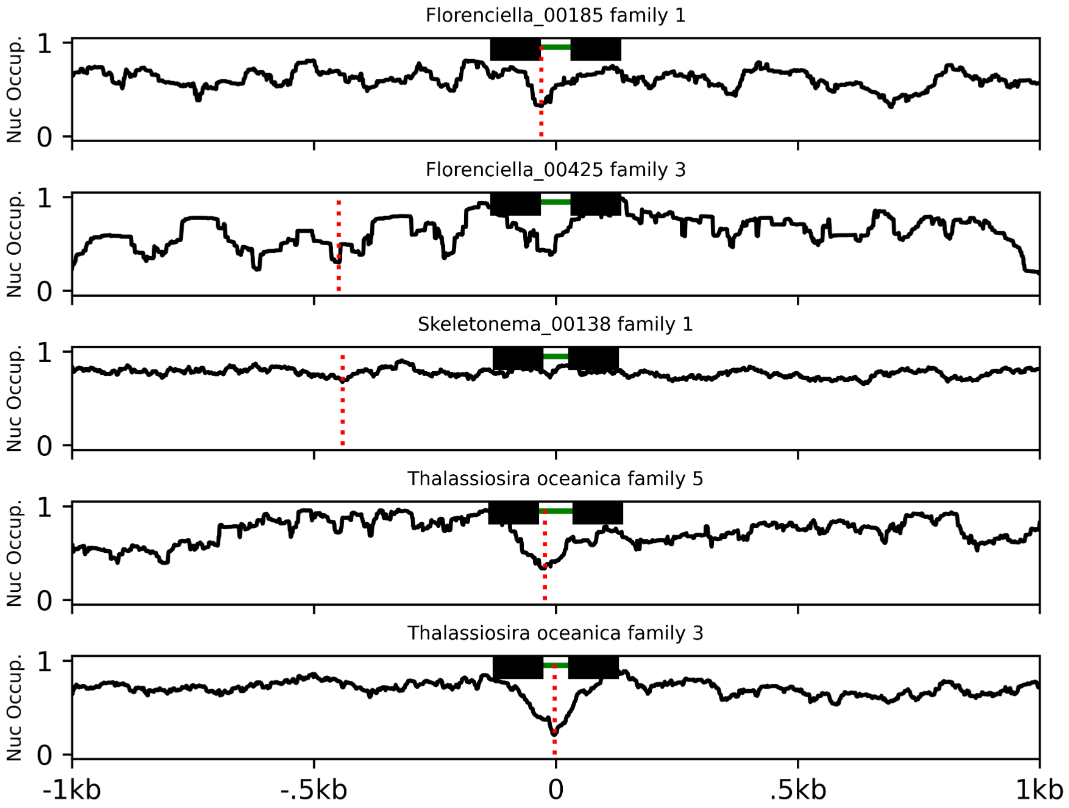
Florenciella_00185 family 1
Florenciella_00425 family 3
Skeletonema_00138 family 1
Thalassiosira oceanica family 5
Thalassiosira oceanica family 3
Nuc Occup.
1
0
-1kb
-.5kb
0
.5kb
1kb

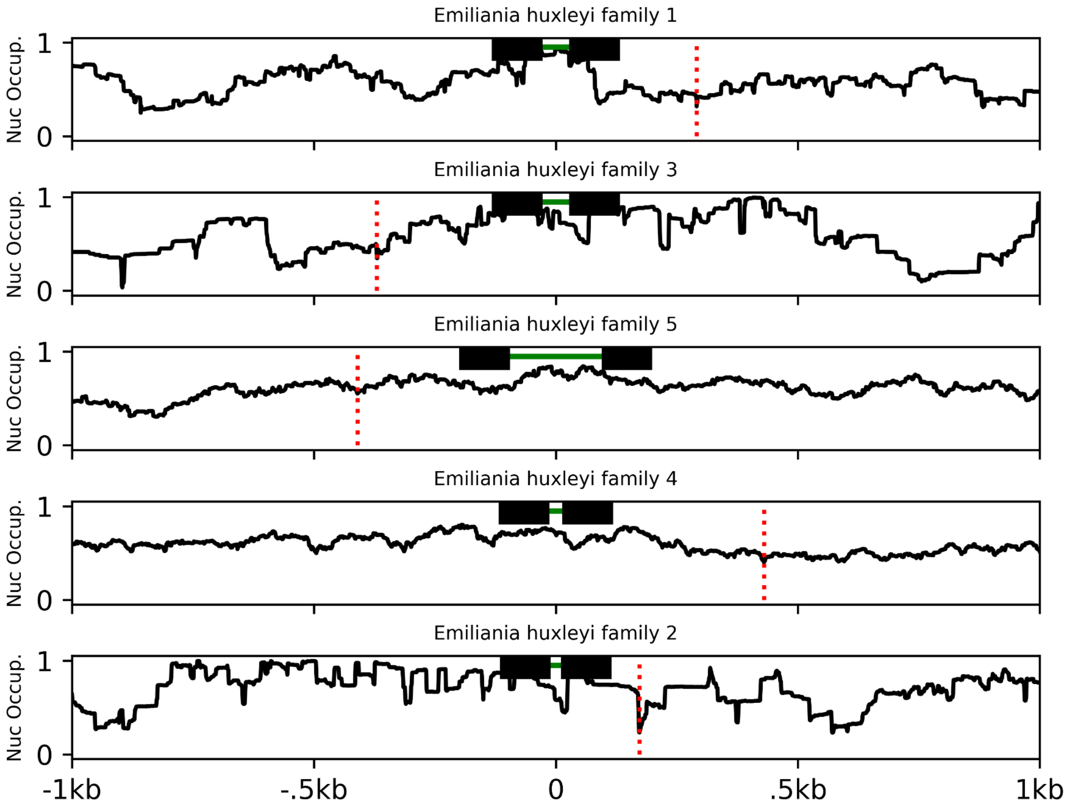
Emiliania huxleyi family 1
Emiliania huxleyi family 3
Emiliania huxleyi family 5
Emiliania huxleyi family 4
Emiliania huxleyi family 2
Nuc Occup.
1
0
-1kb
-.5kb
0
.5kb
1kb

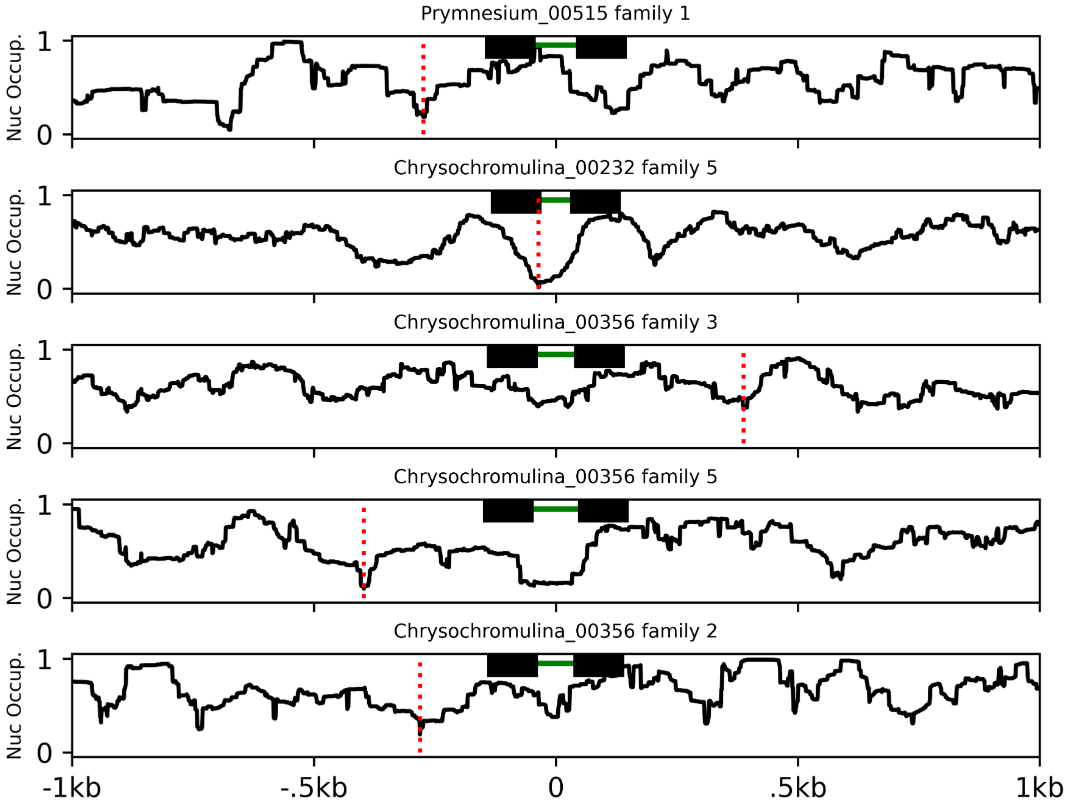
Prymnesium_00515 family 1
Chrysochromulina_00232 family 5
Chrysochromulina_00356 family 3
Chrysochromulina_00356 family 5
Chrysochromulina_00356 family 2
Nuc Occup.
1
0
-1kb
-.5kb
0
.5kb
1kb

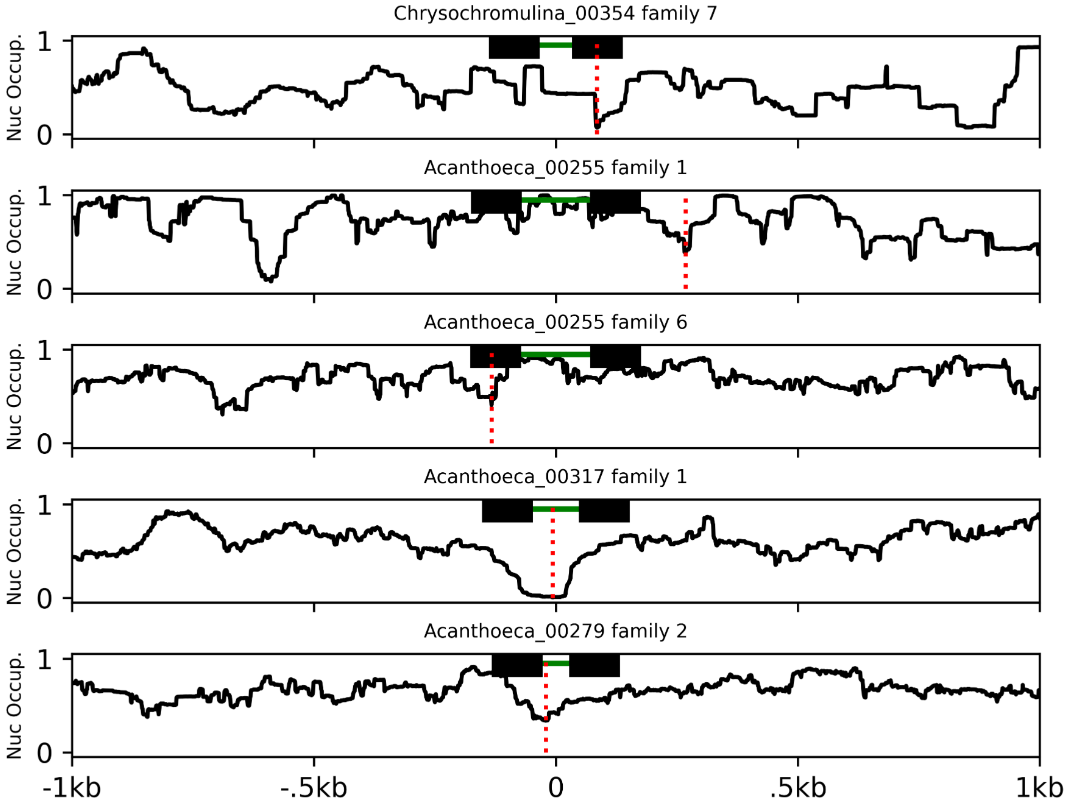

Chrysochromulina_00354 family 7
Acanthoeca_00255 family 1
Acanthoeca_00255 family 6
Acanthoeca_00317 family 1
Acanthoeca_00279 family 2
Nuc Occup.
1
0
-1kb
-.5kb
0
.5kb
1kb

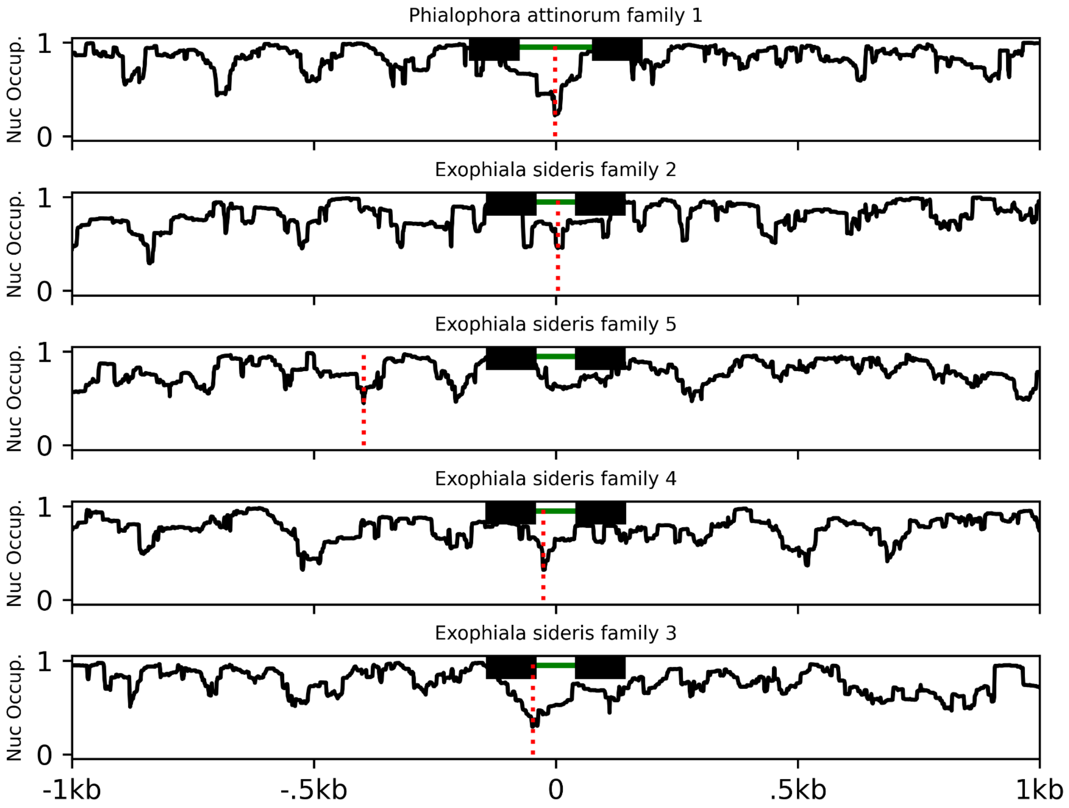

Phialophora attinorum family 1
Exophiala sideris family 2
Exophiala sideris family 5
Exophiala sideris family 4
Exophiala sideris family 3
Nuc Occup.
1
0
-1kb
-.5kb
0
.5kb
1kb

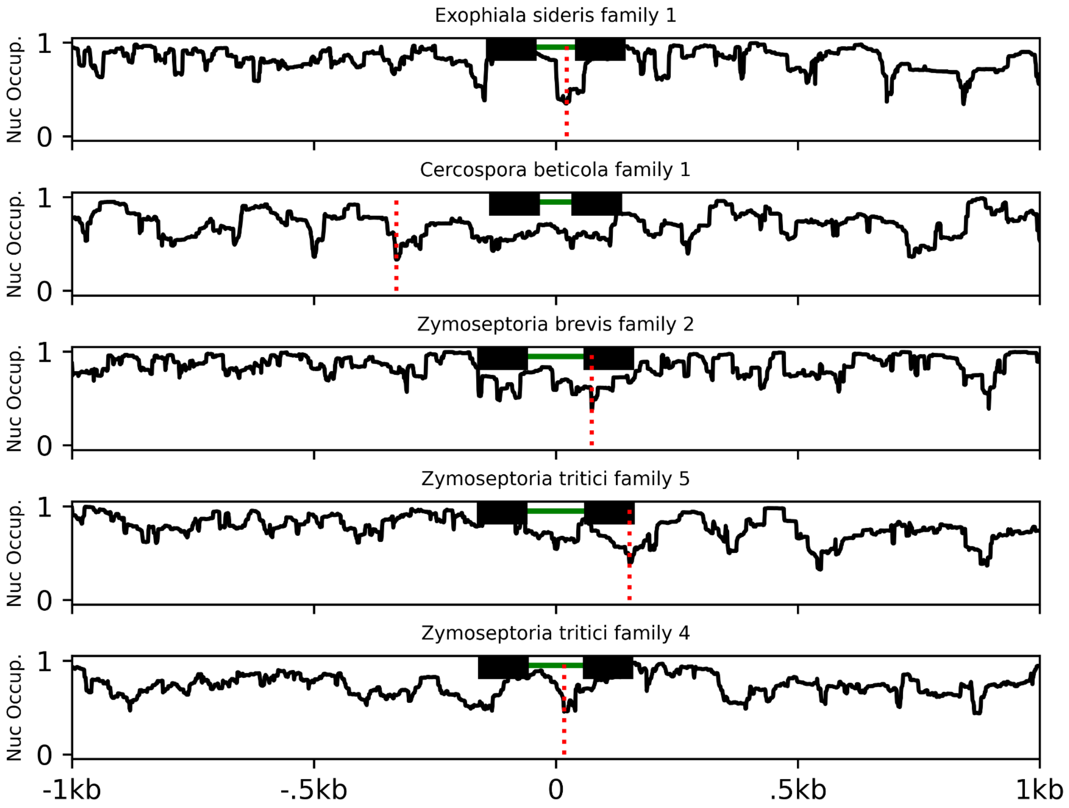
Exophiala sideris family 1
Cercospora beticola family 1
Zymoseptoria brevis family 2
Zymoseptoria tritici family 5
Zymoseptoria tritici family 4
Nuc Occup.
1
0
-1kb
-.5kb
0
.5kb
1kb

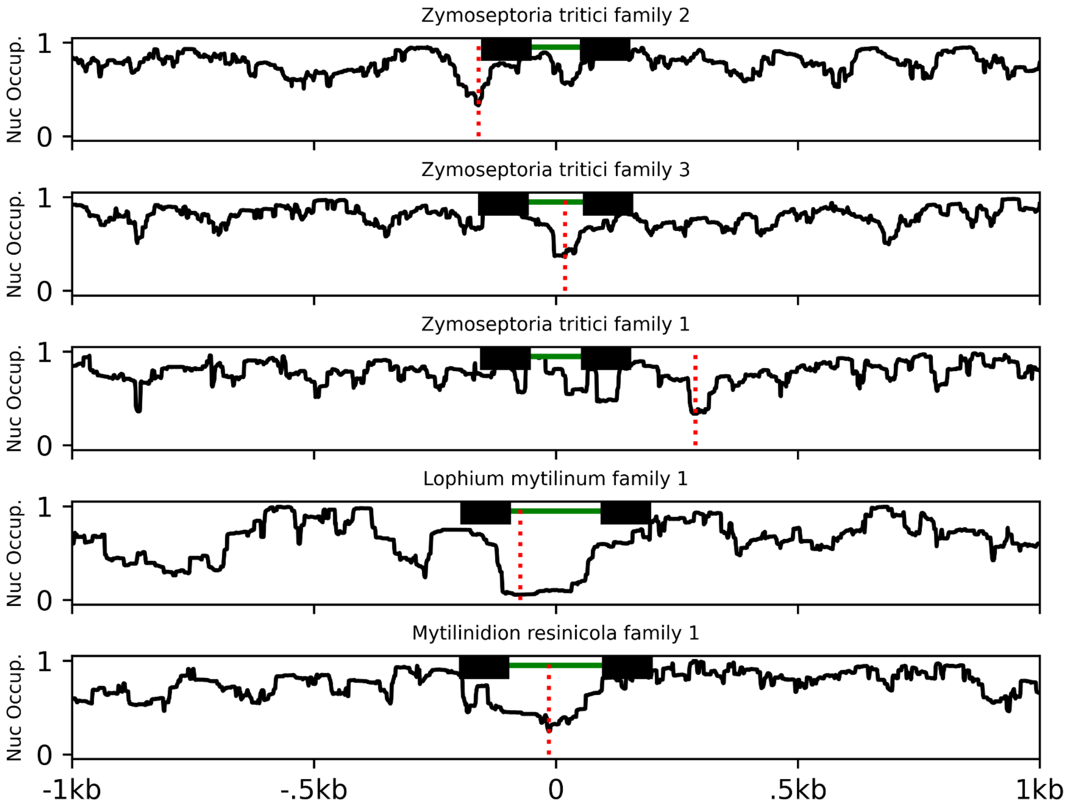

Zymoseptoria tritici family 2
Zymoseptoria tritici family 3
Zymoseptoria tritici family 1
Lophium mytilinum family 1
Mytilinidion resinicola family 1
Nuc Occup.
1
0
-1kb
-.5kb
0
.5kb
1kb

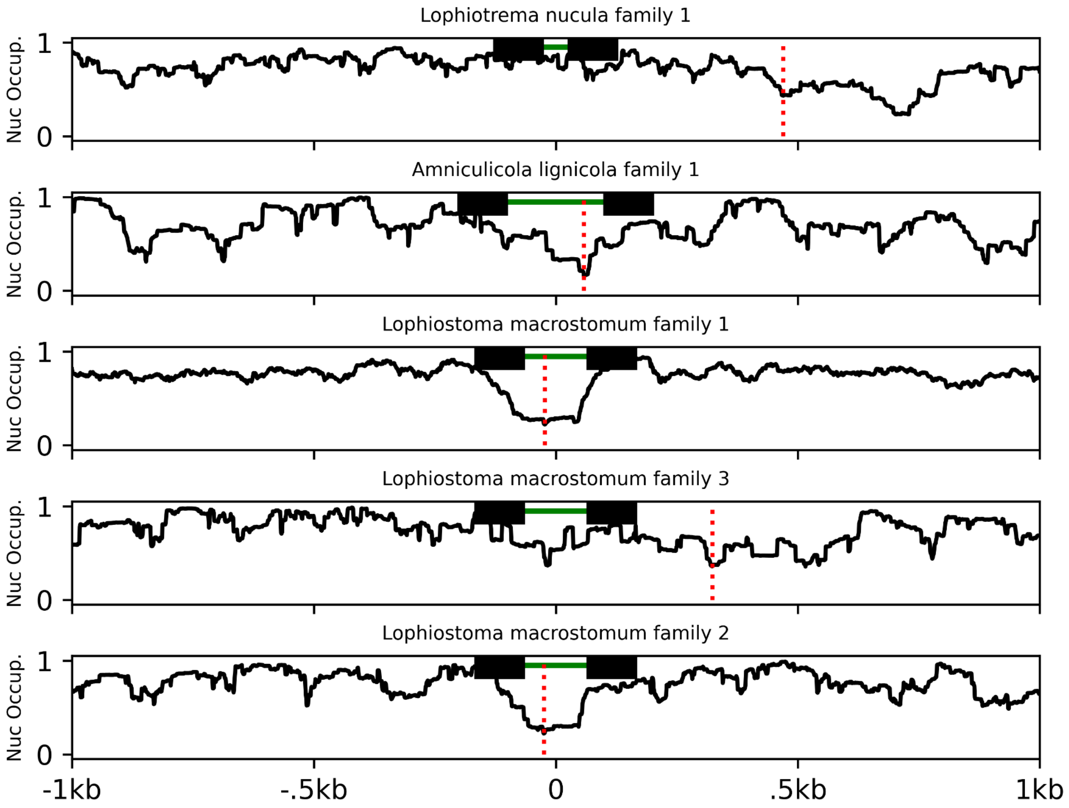
Lophiotrema nucula family 1
Amniculicola lignicola family 1
Lophiostoma macrostomum family 1
Lophiostoma macrostomum family 3
Lophiostoma macrostomum family 2
Nuc Occup.
1
0
-1kb
-.5kb
0
.5kb
1kb

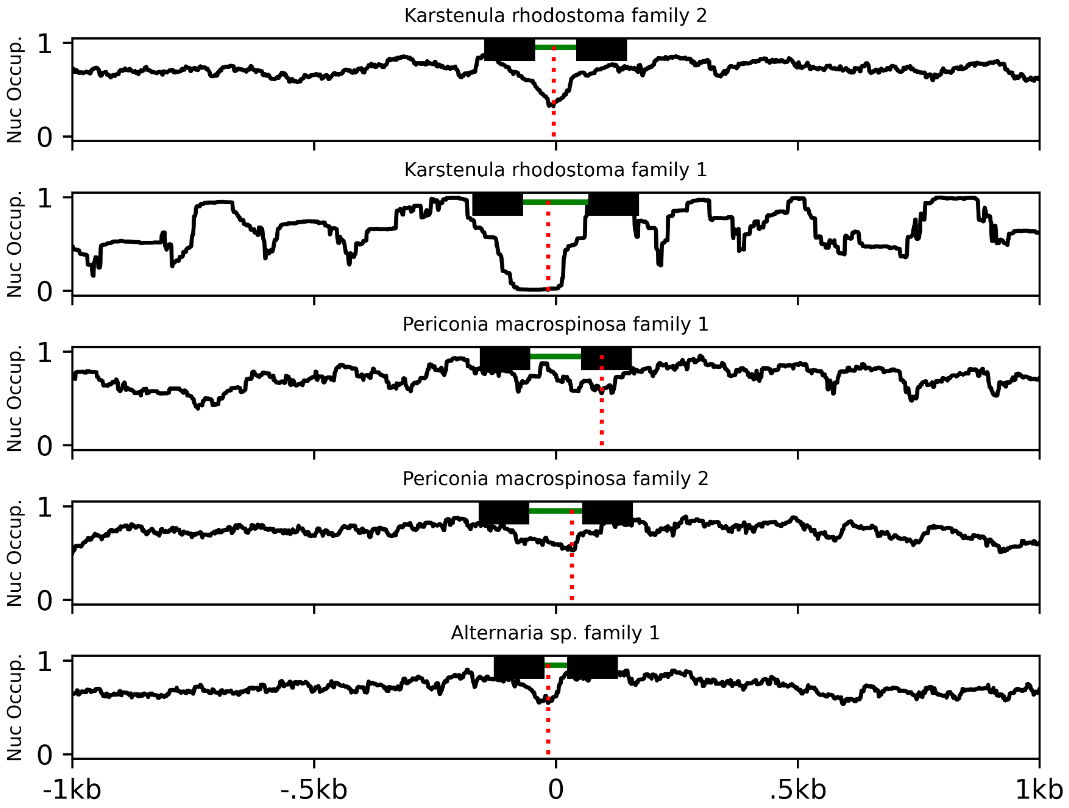
Karstenula rhodostoma family 2
Karstenula rhodostoma family 1
Periconia macrospinosa family 1
Periconia macrospinosa family 2
Alternaria sp. family 1
Nuc Occup.
1
0
-1kb
-.5kb
0
.5kb
1kb

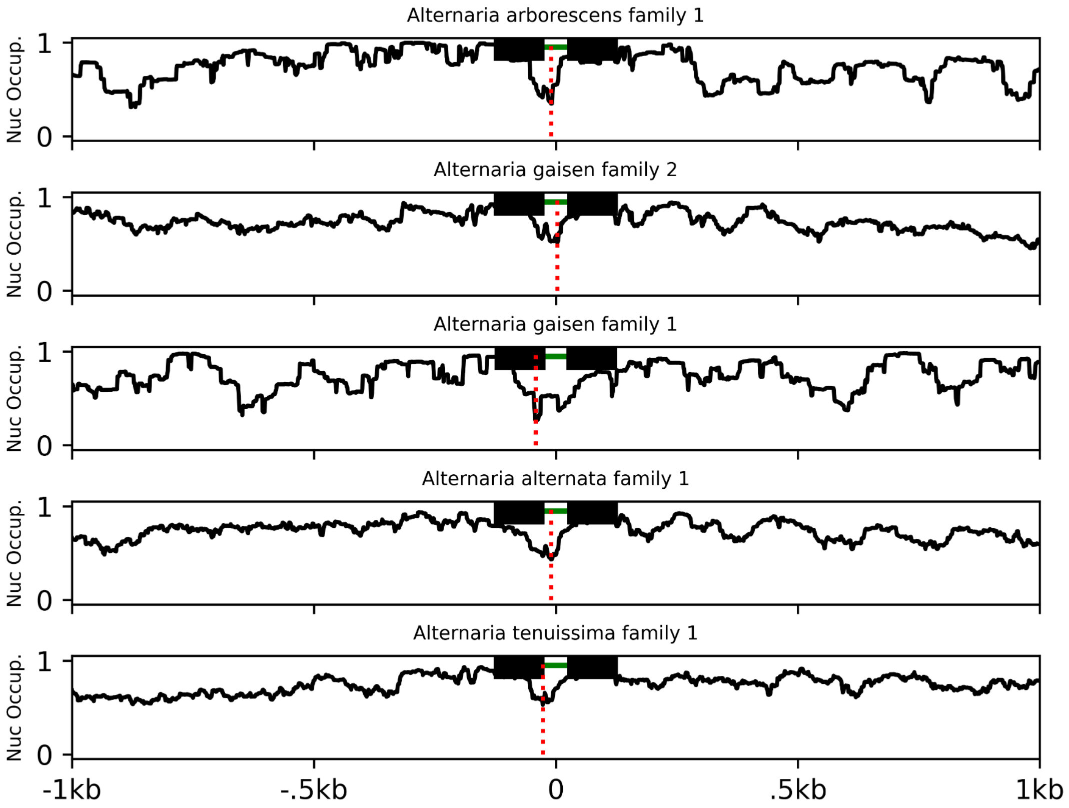

Alternaria arborescens family 1
Alternaria gaisen family 2
Alternaria gaisen family 1
Alternaria alternata family 1
Alternaria tenuissima family 1
Nuc Occup.
1
0
-1kb
-.5kb
0
.5kb
1kb

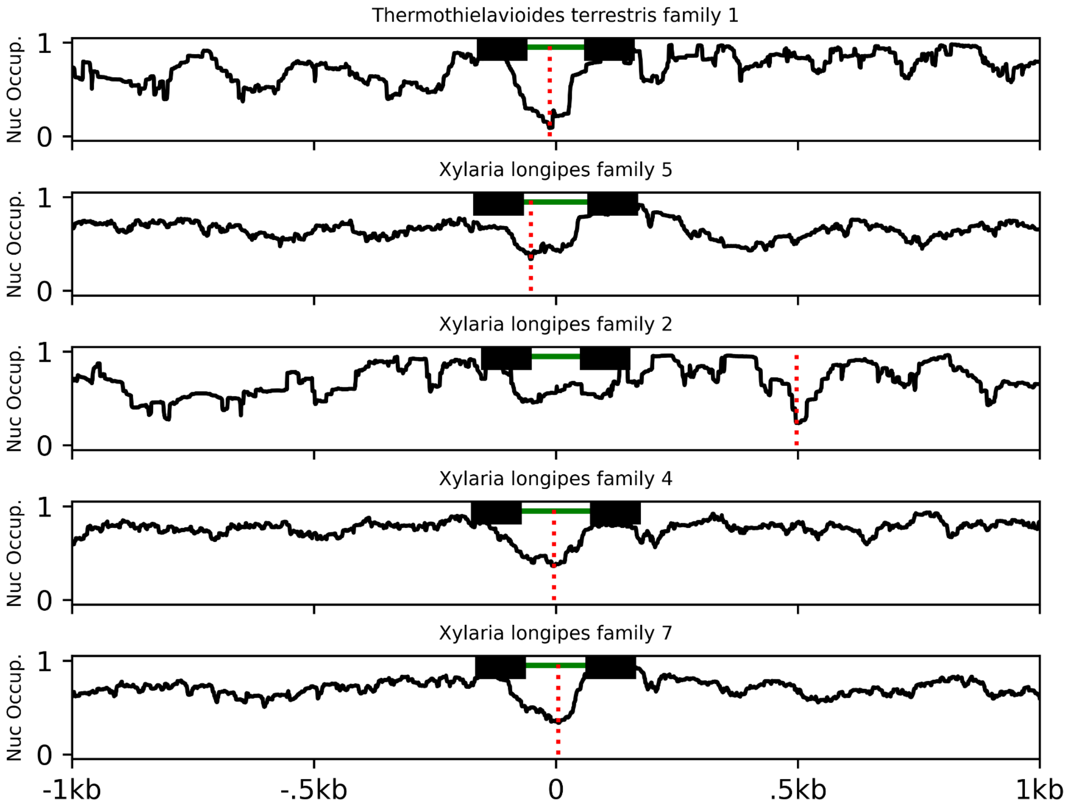

Thermothielavioides terrestris family 1
Xylaria longipes family 5
Xylaria longipes family 2
Xylaria longipes family 4
Xylaria longipes family 7
Nuc Occup.
1
0
-1kb
-.5kb
0
.5kb
1kb

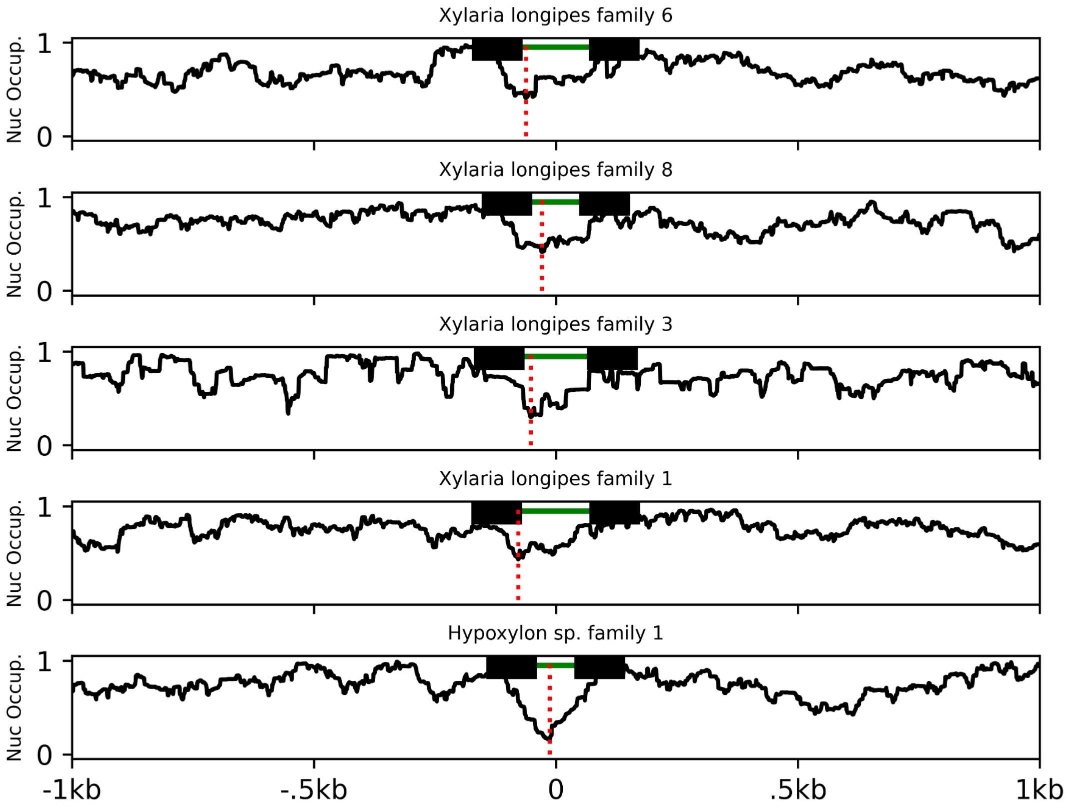

Xylaria longipes family 6
Nuc Occup.
1
0
Xylaria longipes family 8
Nuc Occup.
1
0
Xylaria longipes family 3
Nuc Occup.
1
0
Xylaria longipes family 1
Nuc Occup.
1
0
Hypoxylon sp. family 1
Nuc Occup.
1
0
-1kb
-.5kb
0
.5kb
1kb

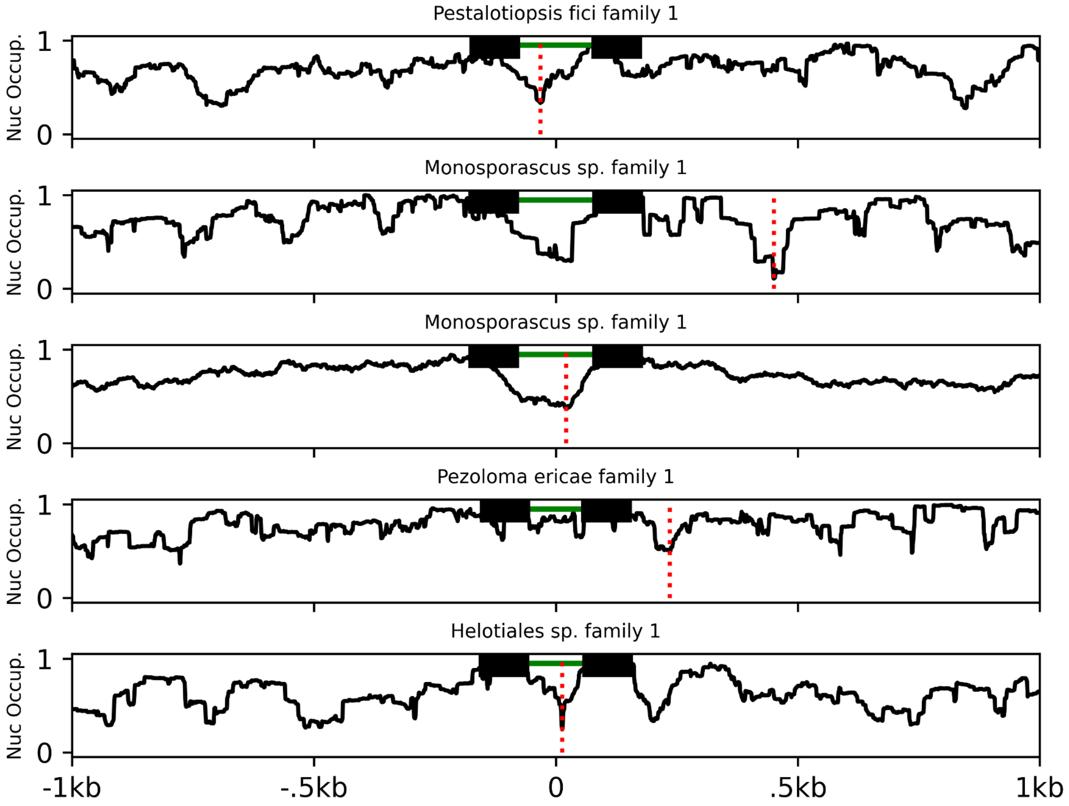

Pestalotiopsis fici family 1
Monosporascus sp. family 1
Monosporascus sp. family 1
Pezoloma ericae family 1
Helotiales sp. family 1
Nuc Occup.
1
0
-1kb
-.5kb
0
.5kb
1kb

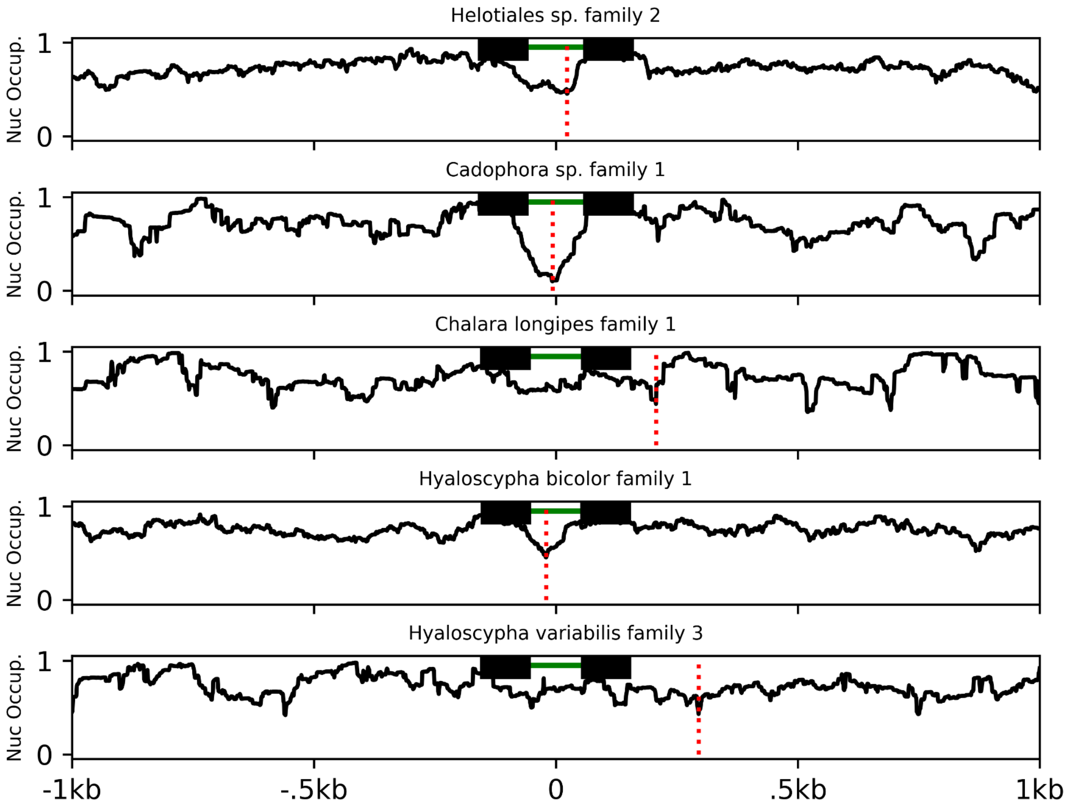

Helotiales sp. family 2
Cadophora sp. family 1
Chalara longipes family 1
Hyaloscypha bicolor family 1
Hyaloscypha variabilis family 3
Nuc Occup.
1
0
-1kb
-.5kb
0
.5kb
1kb

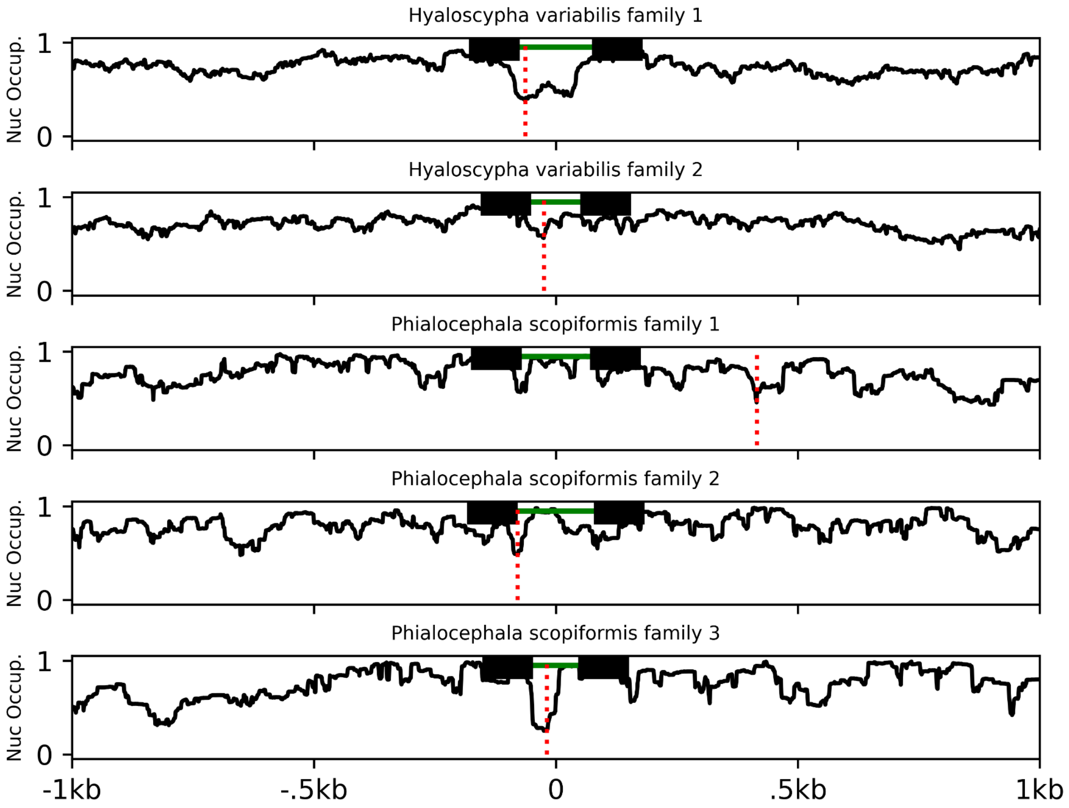
Hyaloscypha variabilis family 1
Hyaloscypha variabilis family 2
Phialocephala scopiformis family 1
Phialocephala scopiformis family 2
Phialocephala scopiformis family 3
Nuc Occup.
1
0
-1kb
-.5kb
0
.5kb
1kb

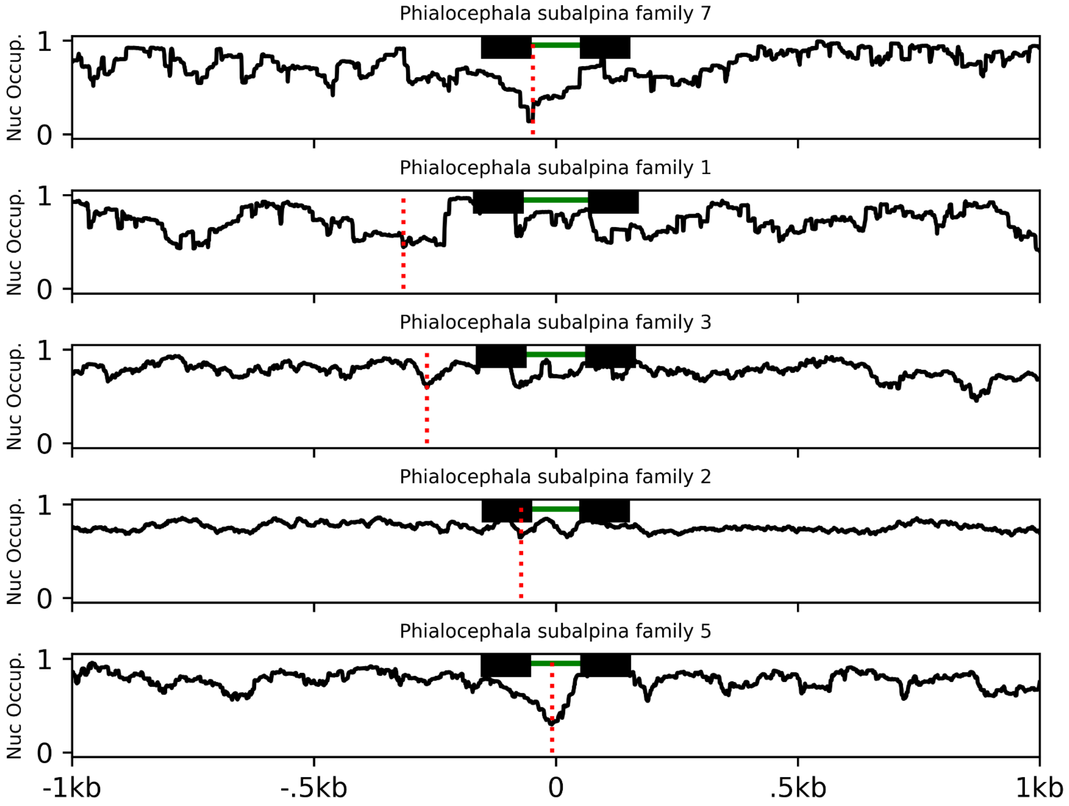

Phialocephala subalpina family 7
Phialocephala subalpina family 1
Phialocephala subalpina family 3
Phialocephala subalpina family 2
Phialocephala subalpina family 5
Nuc Occup.
1
0
-1kb
-.5kb
0
.5kb
1kb

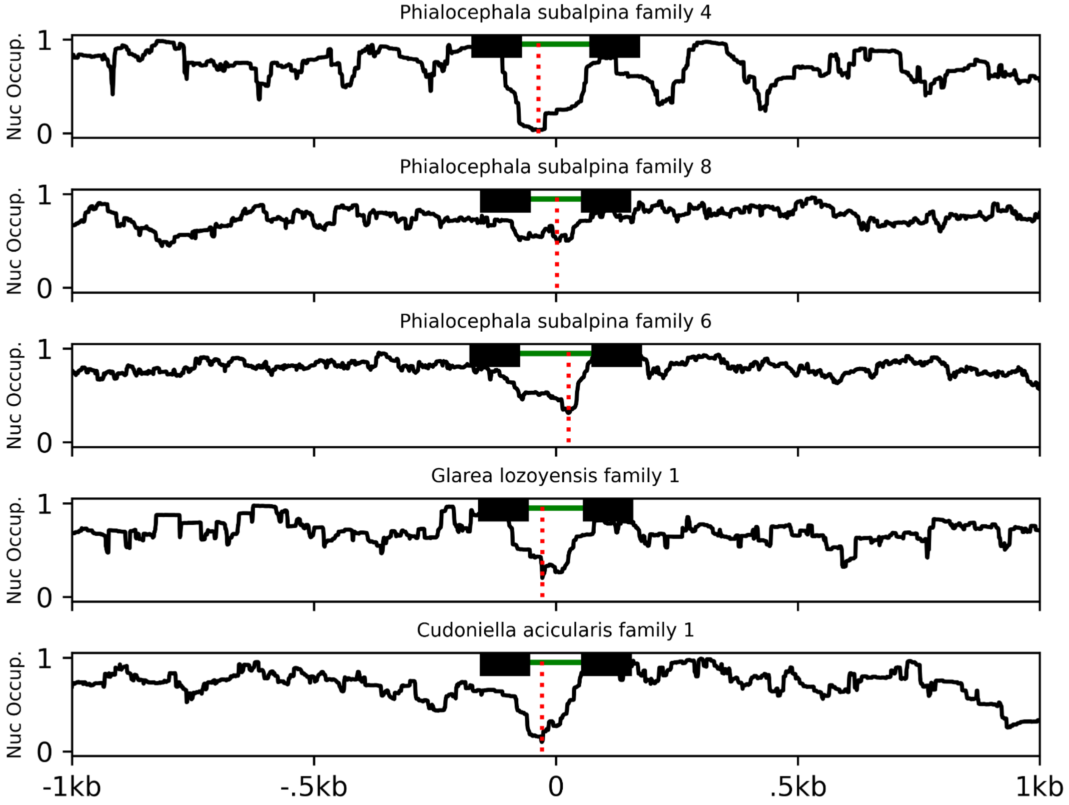
Phialocephala subalpina family 4
Phialocephala subalpina family 8
Phialocephala subalpina family 6
Glarea lozoyensis family 1
Cudoniella acicularis family 1
Nuc Occup.
1
0
-1kb
-.5kb
0
.5kb
1kb

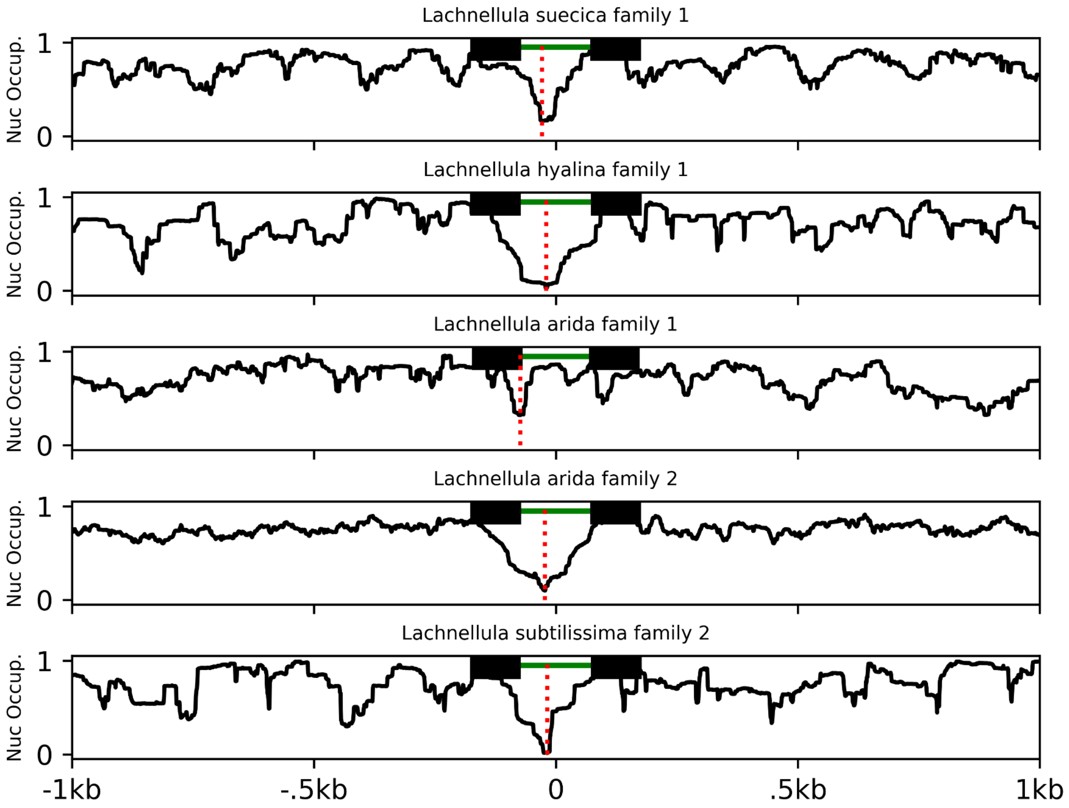

Supplementary Figure 7: Average nucleosome occupancy relative to Introner position across all families in each species. Line plots show the average nucleosome occupancy for all Introners for each family on which we could make nucleosome predictions relative to Introner position. The green bar at the top of each plot represents the Introner and lack boxes represent neighboring exons. The red dotted line represents the position at which we observe the minimum nucleosome occupancy in the given region. Coordinates are shown with reference to Introner position. We find that the lowest trough in nucleosome occupancy for the 2kb region surrounding and containing Introners often exists either near or within Introners in most families, further suggesting that Introners inhabit nucleosome linker regions. In some Introner families, such as those found in Blastocystis sp., predictions are noisy due to either a small sample size on which to make predictions (small families of short Introners) or sequence features inhibiting accurate predictions related to specific species (see section on nucleosome prediction above).

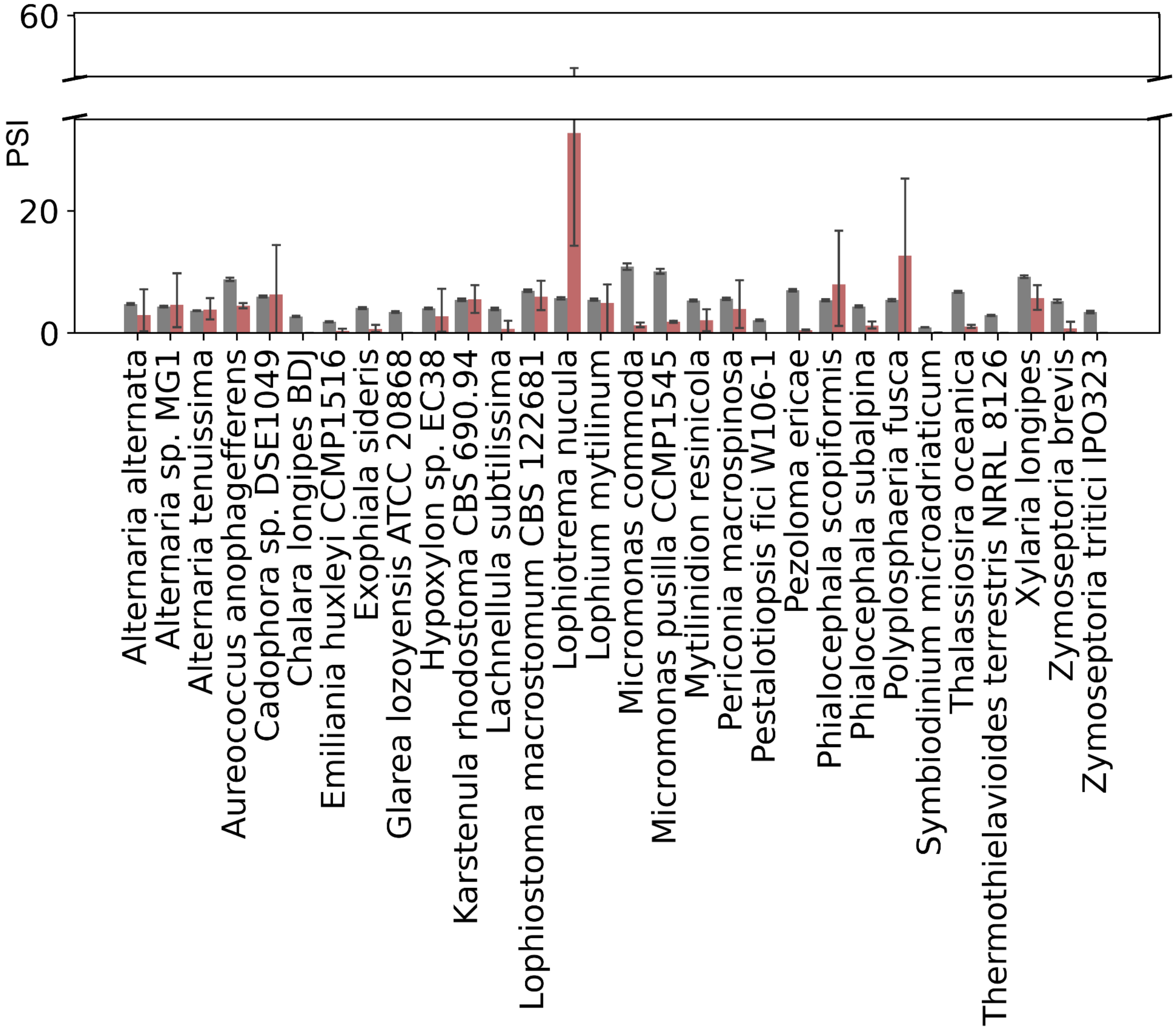

Supplementary Figure 8: **Percent spliced in (PSI) differences between Introners and other Introns.** Bar plots show PSI results for non-Introner introns (gray) and Introners (red) for each species with a Genbank genome and available RNA-seq data. Error bars show 95% confidence intervals. Introners in most species exhibit lower PSI values than other introns, suggesting that they are spliced more efficiently.

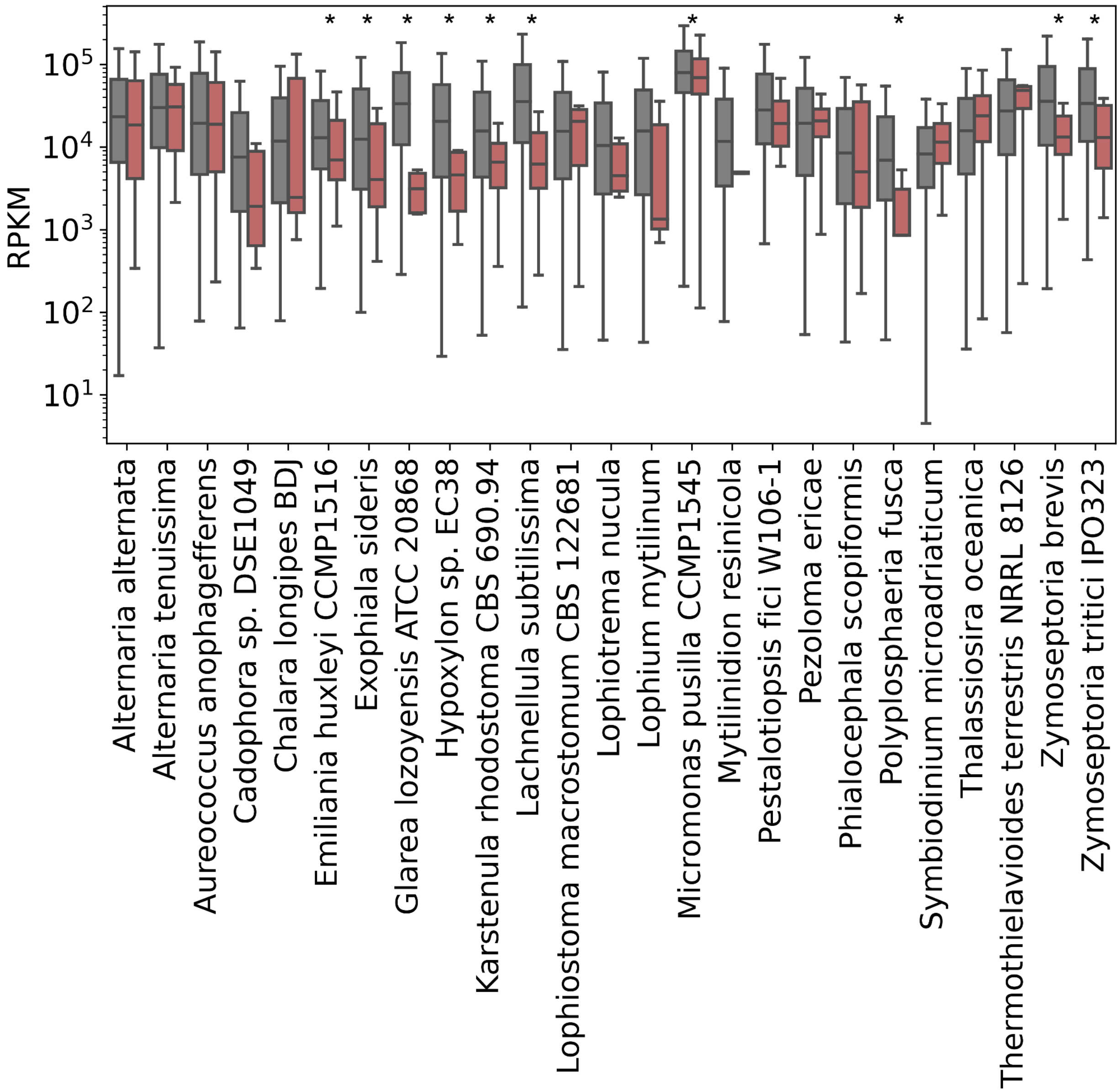

Supplementary Figure 9: **Differential expression between Introner-containing and non-Introner intron containing genes.** Box plots show reads per kilobase of exon per million (RPKM) for non-Introner intron containing genes (gray) and Introner containing genes (red) for each species with a Genbank genome and available RNA-seq data. Introner containing genes are generally more lowly expressed than other Intron containing genes. Asterisks (*) denote statistical significance by Man Whitney U tests.

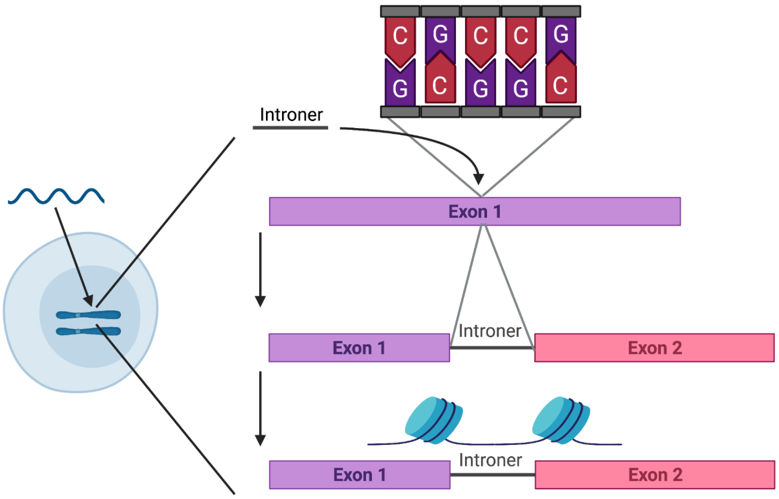

Supplementary Figure 10: Model for intron gain. Foreign DNA containing an Introner enters a species (in this case a unicellular eukaryotic organism) via horizontal gene transfer. The Introner transposes into the new host's germline, inserting preferentially into GC rich regions and causing intron gain upon insertion into an exon. Introners generally inhabit nucleosome linker regions whether they preferentially insert in linker regions or cause a change in nucleosome profiles after insertion.
